# Cell communication and relevant signaling pathways in osteogenesis–angiogenesis coupling

**DOI:** 10.1038/s41413-025-00417-0

**Published:** 2025-04-07

**Authors:** Shuqing Li, Xinjia Cai, Jiahe Guo, Xiaolu Li, Wen Li, Yan Liu, Mengchun Qi

**Affiliations:** 1 Department of Oral & Maxillofacial Surgery, College of Stomatology, North China University of Science and Technology, Tangshan, Hebei China; 2 Central Laboratory, Peking University School and Hospital for Stomatology & National Center for Stomatology & National Clinical Research Center for Oral Diseases & National Engineering Research Center of Oral Biomaterials and Digital Medical Devices & Beijing Key Laboratory of Digital Stomatology & Research Center of Engineering and Technology for Computerized Dentistry Ministry of Health & NMPA Key Laboratory for Dental Materials & National Engineering Research Center of Oral Biomaterials and Digital Medical Devices, Beijing, China

**Keywords:** Bone, Pathogenesis

## Abstract

Osteogenesis is the process of bone formation mediated by the osteoblasts, participating in various bone-related physiological processes including bone development, bone homeostasis and fracture healing. It exhibits temporal and spatial interconnectivity with angiogenesis, constructed by multiple forms of cell communication occurring between bone and vascular endothelial cells. Molecular regulation among different cell types is crucial for coordinating osteogenesis and angiogenesis to facilitate bone remodeling, fracture healing, and other bone-related processes. The transmission of signaling molecules and the activation of their corresponding signal pathways are indispensable for various forms of cell communication. This communication acts as a “bridge” in coupling osteogenesis to angiogenesis. This article reviews the modes and processes of cell communication in osteogenesis-angiogenesis coupling over the past decade, mainly focusing on interactions among bone-related cells and vascular endothelial cells to provide insights into the mechanism of cell communication of osteogenesis-angiogenesis coupling in different bone-related contexts. Moreover, clinical relevance and applications are also introduced in this review.

## Introduction

Osteogenesis, a fundamental biological process, is essential for bone development, remodeling, homeostasis, and fracture healing. Angiogenesis, the formation of new blood vessels from pre-existing ones, plays a pivotal role in this process. Bone, as a highly vascularized tissue, relies on angiogenesis for the transportation of essential nutrients, oxygen, and osteogenic/osteoclastic lineage cells to bone-forming sites.^1–3^ In turn, bone-related cells and signaling molecules tightly regulate angiogenesis.^4^ The interplay between osteogenesis and angiogenesis is intricately coupled, occurring in a synchronized temporal and spatial manner, underscoring their interdependence.^1–3^

Both bone and blood vessels function as endocrine organs, secreting an array of signaling molecules to regulate one another. The coupling of osteogenesis and angiogenesis involves intricate interactions between bone-related cells and vascular endothelial cells (ECs). In the bone microenvironment, cells such as osteoblasts (OBs), osteoclasts (OCs), mesenchymal stem cells (MSCs), macrophages, and immune cells secrete cytokines and growth factors that modulate EC proliferation and differentiation, subsequently influencing angiogenesis.^2^ Similarly, ECs release osteogenic cytokines, termed angiocrine factors, which regulate bone formation and remodeling.^3^ This bidirectional communication forms a complex signaling network that mediates osteogenesis-angiogenesis coupling.

Central to this process is signal transmission between bone and vascular cells, facilitated by diverse modes of cell communication. These include direct interactions such as gap junctions and tight junctions, as well as indirect mechanisms like paracrine, endocrine, and extracellular vesicle-mediated signaling.^2,4^ Other regulatory mechanisms, such as microRNA (miRNA) and epigenetic modifications, also play significant roles.^5^ The biological outcomes of these communications are largely governed by intracellular signaling pathways activated in target cells, linking signal transmission to specific biological responses.

This review explores the modes of cell communication and key signaling pathways involved in osteogenesis-angiogenesis coupling. It delves into their roles in bone development, homeostasis, fracture healing, and bone-related diseases. Furthermore, potential clinical applications based on these mechanisms are discussed, highlighting their relevance to bone tissue engineering and the treatment of bone-related diseases. By synthesizing recent advances, this review provides a comprehensive understanding of cell communication in osteogenesis-angiogenesis coupling, offering new insights into the manipulation of bone formation, regeneration, and disease treatment.

## Cell communication modes and key signaling pathways in osteogenesis-angiogenesis coupling

### Cell communication mechanisms

Cell communication primarily encompasses two fundamental processes: signal conduction and signal transduction.^6^ Signal conduction refers to the production of signaling molecules by donor cells and their transmission to recipient cells. In the context of osteogenesis-angiogenesis coupling, various cell types—such as bone-related cells, immune cells, and vascular cells—participate in this process. The intricate interactions and extensive signaling among these cells significantly amplify the complexity of cell communication. Additionally, intercellular signal transmission relies on distinct communication modes, which not only determine the recipient cells but also influence their subsequent biological responses.

**Fig. 1:**
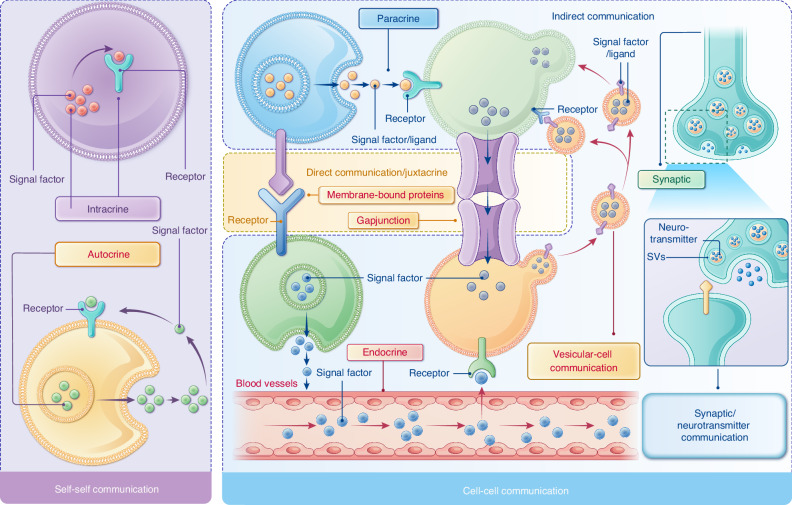
Schematic diagram of different cell communication modes. Among these modes, intracrine and autocrine involve signal transmission within the same cell (self-communication), whereas the other modes facilitate communication between different cells (cell-to-cell communication), either directly or indirectly. Direct cell-cell communication (juxtacrine) occurs through physical contact between two cells, either via ligand-receptor interactions on cell membranes or through intercellular structures like gap junctions and membrane nanotubes, which allow the exchange of diffusible signaling molecules. Meanwhile, indirect cell-cell communication involves the transmission of signaling molecules (ligands) over a certain distance from donor cells to recipient cells. For instance, paracrine signaling affects nearby cells over short distances through diffusion, while endocrine signaling uses blood circulation to transport hormones over long distances to their target cells. Synaptic/neurotransmitter communication involves the release of neurotransmitter from synaptic vesicles (SVs) into synapses, and vesicular-cell communication employs extracellular vehicles (EVs) to deliver signaling molecules to target cells

As an essential step following signal generation in donor cells, intercellular signal transmission establishes a communication bridge between donor and recipient cells. Signal molecules secreted by donor cells reach recipient cells through various mechanisms, including diffusion, blood circulation, direct contact, and intercellular junctions (e.g., gap junction channel (GJC) and other tubular structures).^6^ Based on the mode of signal transmission, cell communication can be classified into several types, including intracrine, autocrine, paracrine, juxtacrine, endocrine, vesicular-cell communication, and synaptic/neurotransmitter communication (Fig. 1).

Following signal conduction, signal transduction occurs. This process involves the interaction between ligands and their receptors, facilitating the transfer of extracellular signals into recipient cells. Intracellular signal transduction then takes place, involving the amplification of these signals through specific signaling pathways. Ultimately, these processes trigger the biological responses of recipient cells, shaping the outcomes of osteogenesis-angiogenesis coupling. Abnormalities in the components of signaling pathways can disrupt intracellular signal transduction, preventing the signals from eliciting appropriate intracellular effects even after reaching the target cells. Such disruptions can lead to the development of diseases.^7^ Therefore, signaling pathways are critical to ensuring proper intracellular transduction within recipient cells.

Both the communication modes for signal conduction among various cell types and the signaling pathways responsible for signal transduction within recipient cells are integral to cell communication in osteogenesis-angiogenesis coupling. A comprehensive understanding of these mechanisms is essential for elucidating their roles in bone formation and vascularization.

### Major cell communication modes in osteogenesis-angiogenesis coupling

Endocrine signaling typically facilitates long-distance communication, exerting widespread effects and connecting different organs throughout the body.^8^ However, in osteogenesis-angiogenesis coupling, cell communication predominantly occurs within localized areas due to the natural proximity between bone-forming cells and ECs. While endocrine-transmitted signal molecules are partially involved, their role in osteogenesis-angiogenesis coupling is relatively limited. Despite the recognition of bone and blood vessels as endocrine organs,^3^ endocrine signaling plays only a minor role in this localized communication process. Similarly, synaptic/neurotransmitter communication is primarily associated with systemic regulation, particularly the neurovascular coupling that integrates nerve signals with vascular responses, rather than facilitating direct signal exchange between bone-forming cells and ECs.^9,10^ In contrast, the primary modes of communication in osteogenesis-angiogenesis coupling are paracrine, juxtacrine, autocrine, and extracellular vesicle (EV)-mediated communication. These modes work in concert to orchestrate the intricate interactions between cells, forming a harmonious “symphony” that directs the coupling of osteogenesis and angiogenesis.

#### Paracrine

Paracrine communication is one of the most common modes in osteogenesis-angiogenesis coupling. In this mode, both bone-related cells and ECs secrete signaling molecules to the extracellular environment to affect both osteogenesis and angiogenesis. ECs produce a range of vascular-derived factors, including bone morphogenetic protein (BMP)-2/4/7, insulin-like growth factor (IGF), and parathyroid hormone, which regulate the biological activity of bone-related cells, including OBs, OCs, and bone marrow-derived mesenchymal stem cells (BMSCs).^3^ Conversely, factors secreted by OBs, OCs, and BMSCs, such as vascular endothelial growth factor (VEGF) A and platelet-derived growth factor (PDGF)-BB,^3,11^ act on ECs to influence their migration, proliferation, and angiogenic capabilities.

This reciprocal exchange of signals establishes a complex intercellular communication network that bridges osteogenesis and angiogenesis. Beyond bone-related cells and ECs, other cells in the bone microenvironment, such as macrophages and immune cells, also actively contribute to the coupling of osteogenesis and angiogenesis through paracrine signaling.

#### Juxtacrine

Juxtacrine communication involves direct cell-to-cell contact, requiring physical proximity between donor and recipient cells. It occurs through two primary mechanisms: gap junctions and direct membrane contact.^12,13^

Gap junctions allow signaling molecules to pass directly between adjacent cells through intercellular channels without exposure to the extracellular environment. These channels, often formed by connexin proteins like connexin 43 (Cx43/Gja1), facilitate cytoplasmic signal exchange, playing a pivotal role in osteogenesis-angiogenesis coupling. Cx43 is widely expressed in bone-related cells (e.g., OBs, osteocytes, chondrocytes, and OCs) and vascular cells, where it regulates critical cellular functions.^14–17^ For instance, osteocytes embedded in the bone matrix use GJCs to transmit biochemical signals, such as hormones and mechanical stimuli, to OBs and OCs, modulating their activity.^18^ In ECs, GJCs affect vascular permeability and angiogenic potential.^15^ Similarly, P channels formed by pannexins, such as pannexin-3 in OBs and pannexin-1 in ECs, contribute to osteogenesis and angiogenesis through processes like Ca²⁺ signaling and ATP release, respectively.^19,20^ Despite these findings, the precise mechanisms by which gap junctions coordinate osteogenesis-angiogenesis coupling remain underexplored.

Direct membrane contact, this form of juxtacrine signaling relies on the interaction between membrane-bound ligands on donor cells and their receptors on recipient cells, activating intracellular signaling pathways. For example, the NOTCH signaling pathway, mediated by such direct interactions, influences both osteogenesis and angiogenesis.^21^ Similarly, WNT signaling, such as wnt1 derived from BMSCs, has been shown to trigger intracellular effects through membrane contact.^22^ These interactions integrate classical signaling pathways to coordinate bone and vascular processes.

Adherens and tight junctions provide structural stability for cell-cell interactions, facilitating membrane protein-mediated communication.^23,24^ Beyond structural support, adherens junction proteins can activate intracellular pathways, contributing to signaling in osteogenesis and angiogenesis coupling.^25,26^ While their specific roles remain to be fully elucidated, these junctions likely play a supportive role in maintaining effective juxtacrine communication between cells.

#### Autocrine

Autocrine communication refers to a self-regulatory mechanism where signaling molecules produced by a cell act on the same cell, activating intracellular signaling pathways and eliciting specific biological effects. This mode of communication plays a significant role in osteogenesis-angiogenesis coupling.

For instance, VEGF, primarily known for its paracrine role, is secreted by OBs and other bone-related cells to interact with receptors on adjacent ECs, regulating their differentiation and angiogenesis. Meanwhile, vascular endothelial growth factor receptor 2 (VEGFR2) is also expressed on osteogenic lineage cells that produce VEGF.^27^ This suggests that OB-derived VEGF not only influences ECs through paracrine mechanisms but also regulates the differentiation and activity of OBs themselves via autocrine signaling.^28,29^

#### EVs and vesicular signaling

EVs are vital mediators of cell-cell communication, carrying signaling molecules such as proteins, genes, miRNAs, and other bioactive materials to regulate biological processes, including osteogenesis and angiogenesis. Exosomes, the most studied EVs, are nanoscale vesicles (30–50 nm) with a bilayer lipid membrane originating from multivesicular endosomes. In the bone microenvironment, exosomes are secreted by OBs,^30,31^ OCs,^32^ ECs,^33^ macrophages, and other immunocytes.^34,35^ They play diverse roles in osteogenesis-angiogenesis coupling. Vascular endothelial cell-derived exosomes (EC-exos) regulate the differentiation of OBs and osteoclast precursors, inhibit bone marrow macrophage differentiation, and prevent excessive bone resorption, thereby reducing osteoporosis.^36^ Endothelial progenitor cell-derived exosomes, enriched in long-non-coding-RNA (lncRNA)-MALAT1, these exosomes bind to miRNA124 to promote osteoclast precursor differentiation and recruitment, enhancing bone repair through controlled resorption.^37^ M2 macrophage- and BMSC-derived exosomes transmit signaling molecules to target cells, influencing proliferation, migration, and differentiation, thus impacting osteogenesis-angiogenesis coupling.^34,38^

Migrasomes, recently discovered EVs larger than exosomes (500–3 000 nm), are produced by migrating monocytes along retraction fibers. As these fibers break, migrasomes are released into the extracellular environment and absorbed by surrounding cells. Unlike exosomes, migrasomes are rich in angiogenesis-related factors such as VEGFA and C-X-C motif chemokine ligand (CXCL) 12, potentially promoting angiogenesis.^39,40^ Although their roles in osteogenesis-angiogenesis coupling remain unclear, they are likely involved in this crosstalk. In addition to exosomes and migrasomes, other EVs may contribute to vesicular signaling in osteogenesis-angiogenesis coupling, highlighting the complexity and potential of EV-mediated communication in bone biology.

##### Extracellular matrix (ECM)- and integrins-mediated signaling

In addition to the aforementioned cell communication modes, the ECM plays a pivotal role in osteogenesis-angiogenesis coupling by storing signaling molecules like transforming growth factor β (TGF-β), which are released during bone resorption or remodeling.^41,42^ These ECM-derived signals interact with specific transmembrane receptors on target cells, primarily integrins, to activate intracellular signaling pathways.

Integrins are Ca^2+^ or Mg^2+^-dependent cell adhesion molecules formed by α and β subunits heterodimers. Acting as transmembrane proteins, they mediate ECM-cell communication. For instance, the absence of bone morphogenetic protein receptor 2 in ECs promotes integrin β1 expression, enhancing cell adhesion and facilitating TGF-β activation from its latent fibrillin-bound depots.^43^ However, further studies are needed to fully elucidate the interactions between integrins and ECM-derived signals

Beyond biochemical signaling, integrins also sense mechanical stimuli and ECM physical properties like stiffness, triggering conformational changes that regulate cellular activities. For example, integrin β3 in OCs and their precursors responds to mechanical stimuli, influencing OC growth and differentiation, ultimately determining their fate.^44^ Integrins also mediate cell-cell communication by participating in various mechanisms, such as adherens junctions, gap junctions, and vesicular transport. For example, integrins interact with VE-cadherin, which maintains vascular integrity and limits EC proliferation. This interaction influences cell migration and involves protein tyrosine phosphatase 1B.^45^ Furthermore, Integrins interact with gap junction proteins to regulate cell adhesion and intercellular communication.^46,47^ Studies have shown that Integrins are involved in the endocytosis of EVs, including exosomes, facilitating signal exchange between cells.^48^ Additionally, integrins play a role in intracellular signal transduction. For example, integrin αvβ_3_ participates in the signal transduction of VEGFR2 signaling, affecting migration and angiogenic ability of ECs.^49^

This review summarized the expression and function of different integrins in different cells involved in osteogenesis and angiogenesis, as shown in Table 1.

**Table 1:** Expression and function of integrins in osteogenic and angiogenic cells

Cell type	Integrin	Influence mediated by integrin	Ref.
OBs	α_5_β_1_	Cell adhesion	^318^
BMSCs	α_5_β_1_	osteogenic differentiation, adhesion	^318^
ECs	α_v_β_3_	angiogenesis, adhesion	^49,319^
	β_1_	immigration	^46^
OCs and OC precursors	β_3_	differentiation	^44^
Neutrophil	β_2_	assistant the influence of recruitment made by TRPC6 channel	^47^

### Key signaling pathways in osteogenesis-angiogenesis coupling

Signaling molecules are transmitted to target cells through various modes of cell communication. Once these molecules are recognized by the target cells and bind to their specific receptors, they initiate a cascade of intracellular events through signal transduction. This process activates specific intracellular signaling pathways, inducing a range of biological responses, such as cell proliferation, survival, differentiation, and apoptosis. These cellular processes collectively regulate the coupling between osteogenesis and angiogenesis.

#### Crucial signaling pathways mediated by paracrine

##### VEGF pathway

The VEGF signaling pathway is crucial in osteogenesis-angiogenesis coupling. VEGF binds to its receptor VEGFR via paracrine signaling, activating several downstream pathways, including the phosphoinositide 3-kinase (PI3K)/protein kinase B (Akt), protein kinase C /mitogen-activated protein kinase (MAPK), and p38/MAPK pathways. These pathways collectively regulate migration, survival, proliferation, and angiogenesis of ECs.

For example, VEGF promotes ECs migration, survival, proliferation, and angiogenesis through the PI3K/Akt signaling pathway, with the p110-alpha subtype of PI3K playing a critical role in this process.^6,50,51^ VEGF-activated phospholipase C-γ, an upstream regulator of MAPK signaling, initiates this pathway to drive EC proliferation,^52^ while phospholipase C-β2 regulates vascular permeability.^53^ The MAPK pathway also contributes to EC filopodia formation, a key step in vascular sprouting.^54^ Additionally, Cdc42 is a crucial mediator of VEGF signaling, activating the p38/MAPK pathway to promote EC migration.^55^ Knockout of Cdc42 results in abnormal vascular structures, reduced sprouting, and lethality in mice.^56^ VEGF further regulates angiogenesis and vascular permeability through endothelial nitric oxide synthase, a downstream effector controlled by phospholipase C-γ and PI3K/Akt signaling.^57,58^ Focal adhesion kinase is also modulated by VEGF signaling, further supporting angiogenesis.^59^

VEGF signaling interacts with other pathways to regulate angiogenesis. For instance, the angiopoietin1/Tie2 signaling pathway collaborates with VEGFR to maintain vascular integrity,^60^ while VEGFB prevents excessive angiogenesis by inhibiting fibroblast growth factor 2 (FGF2)/fibroblast growth factor receptor 1 (FGFR1) signaling.^61^ VEGF also facilitates osteogenesis-angiogenesis coupling. During alveolar ossification, VEGFA co-localizes with osteogenic factors such as runt-related transcription factor 2 (Runx2) and Osterix (Osx), thereby regulating OBs differentiation.^62,63^ VEGFA stimulates ECs to upregulate BMP-2 expression, enhancing osteogenesis.^64^ Additionally, the VEGFC and VEGFD influence the formation of OCs, further contributing to osteogenesis-angiogenesis coupling.^65^

##### Hypoxia-inducible factor (HIF)-1α pathway

Hypoxia affects angiogenesis and osteogenesis via HIFs, particularly HIF-1α. Under normoxic conditions, prolyl hydroxylases continuously hydroxylate HIF-1α, marking it for ubiquitination by the E3 ligase von Hippel-Lindau protein, followed by proteasomal degradation.^66^ However, during hypoxia, However, during hypoxia, the activity of prolyl hydroxylases is inhibited due to limited molecular oxygen availability, leading to the stabilization and accumulation of HIF-1α, which is subsequently translocated into the nucleus. Once in the nucleus, HIF-1α forms a heterodimer with HIF-1β and binds to hypoxia response elements on the promoters of target genes, including VEGF. This induces the transcription of VEGF and other genes. Elevated VEGF then binds to its receptors on ECs, stimulating angiogenesis. HIF-1α-regulated VEGF expression forms a crucial signaling axis that facilitates communication among OBs, OCs, and ECs, thereby coupling osteogenesis and angiogenesis.

In OBs, hypoxia or other external stimuli increase HIF-1α expression, promoting the production and secretion of VEGFA, which enhances angiogenesis in ECs through a paracrine mechanism.^67,68^ Conversely, the loss of HIF-1α in OBs reduces angiogenesis in long bones.^68^ Both HIF-1α and VEGFA play pivotal roles in bone formation.^68,69^ Furthermore, high HIF-1α expression in type H ECs promotes angiogenesis in type H capillaries, which, in turn, further enhances osteogenesis.^70^

##### Bone morphogenetic proteins (BMPs) and related pathways

BMPs, a subfamily of the TGF-β superfamily, are purified from demineralized bone matrix and play important roles in regulating OB activities.^71^ BMPs exhibit a strong capacity to induce bone formation.^72,73^ TGF-β, of which 80%–90% is represented by TGF-β1, is stored in the bone matrix in a latent state.^74^ During osteoclast-driven bone resorption, both BMPs and TGF-β1 are released and activated, subsequently fulfilling their roles in bone formation and remodeling.^75^

As members of the TGF-β superfamily, BMPs, and TGF-β share similar classical signaling pathways, including TGF-β/BMP ligands, type I and type II receptors, and downstream effectors such as small mother against decapentaplegic (Smad) proteins. The core signal cascade of these pathways is highly conserved, with differences primarily in ligand and receptor specificity.^76^ In classical signaling, TGF-β activates Smad2/3, while BMPs activate Smad1/5/8/9 upon receptor binding. Smad4, a co-mediating Smad, connects both pathways by binding to activated receptor-Smads and translocates into the nucleus to regulate downstream gene expression. Smad6/7, as inhibitory Smads, suppress receptor-Smad activation in both pathways.^77^ In addition to the classical pathway, TGF-β/BMP signaling interacts with various other pathways, including the Wnt, PI3K/Akt, NOTCH, and MAPK pathways, further diversifying its regulatory mechanisms.^77,78^ Both TGF-β1 and BMPs influence the activities of OBs and OCs, thus regulating osteogenesis.^78–81^ Importantly, TGF-β/BMP signaling pathways also mediate osteogenesis through angiogenesis, contributing to osteogenesis-angiogenesis coupling. This intricate interplay highlights the role of TGF-β/BMP pathways in bridging bone formation and vascular development.^82–84^

##### Wnt/β-catenin pathway

The Wnt signaling pathways are categorized into two types: canonical (Wnt/β-catenin) and noncanonical (non-β-catenin-dependent). Among them, the Wnt/β-catenin pathway plays a pivotal role in osteogenesis-angiogenesis coupling. Activation of this pathway requires the binding of paracrine Wnt proteins to Frizzled receptors and low-density lipoprotein-related receptor 5/6 co-receptors, forming a complex. This activates the cytoplasmic protein Disheveled, which inhibits glycogen synthase kinase-3β from degrading β-catenin. The stabilized β-catenin accumulates in the cytoplasm and translocates to the nucleus, where it interacts with T cell factor/lymphoid enhancer factor to activate the expression of downstream target genes.^85^

The Wnt/β-catenin signaling pathway directly regulates bone formation through promoting BMSCs differentiation into OBs and modulating OBs function.^86^ For instance, Wnt16 activates the canonical Wnt pathway in OBs, inducing the expression of osteoprotegerin (OPG), which inhibits OCs formation and reduces bone resorption.^87,88^ Additionally, Wnt/β-catenin signaling enhances angiogenesis in ECs and endothelial progenitor cells (EPCs), which, in turn, stimulates BMSCs osteogenic differentiation and accelerates bone formation,^89–91^ thereby establishing a coupling between osteogenesis and angiogenesis.

Beyond the canonical pathway, some Wnt proteins also regulate osteogenesis via noncanonical Wnt signaling pathways. For example, osteoblast-derived Wnt4 activates Wnt signaling in OCs and their precursors independently of β-catenin. It suppresses OCs formation and bone resorption by inhibiting nuclear factor-kappa B (NF-κB) activation mediated by TGF kinase 1.^92^ Wnt5a, binding to the receptor protein tyrosine kinase-like orphan receptor 2 expressed on OCs, promotes OCs formation through the Wnt/c-Jun N-terminal kinase pathway.^93^ Wnt16 inhibits receptor activator of nuclear factor-κB ligand (RANKL)-induced NF-κB activation, thereby suppressing OCs formation.^94^ Wnt10 promotes OBs formation and affects bone mass, further underscoring its role in osteogenesis.^95,96^

While noncanonical Wnt signaling pathways have been shown to regulate bone cells, the specific mechanisms by which they influence osteogenesis-angiogenesis coupling require further investigation. This highlights the need for additional studies to elucidate the roles of different Wnt ligands in this complex process.

##### Fibroblast growth factor (FGF) signaling pathway

The FGF signaling pathway is a critical regulator of various physiological processes, including osteogenesis and angiogenesis.^97^ The FGF family comprises more than 20 proteins, which activate downstream signaling by binding to tyrosine kinase receptors, known as FGF receptors. These ligands mediate signaling through different modes of communication: six FGFs function via intracrine, autocrine, or paracrine modes, while the FGF15/19 subfamily, also known as hormone-like FGFs, operates via endocrine signaling.^98^ Paracrine FGFs bind their receptors with the assistance of heparin or heparan sulfate, while endocrine FGFs require Klotho proteins as cofactors. Despite the diversity of communication modes, most FGF ligands play vital roles in osteogenesis-angiogenesis coupling. For instance, FGF9 is critical for both angiogenesis and osteogenesis during long bone repair. Its pro-angiogenic effects are mediated, at least in part, by the regulation of VEGFA.^99^ FGF18, however, promotes osteogenesis but not angiogenesis, as injury-induced angiogenesis remains unimpaired in *FGF*^+/-^ mice.^100^ This suggests that individual FGFs may exhibit specific and independent roles. FGF2 interacts with FGFR1 through paracrine signaling and works in conjunction with prostaglandin E2 to initiate angiogenesis.^101,102^ Other FGF ligands may also regulate osteogenesis-angiogenesis coupling. FGF11 interacts with HIF-1α proteins via intracrine signaling. It stabilizes HIF-1α by inhibiting proteasomal degradation, thereby enhancing hypoxia responses and promoting angiogenesis.^103,104^ Additionally, hypoxia-induced FGF11 stimulates OC-mediated bone resorption, and its expression is dependent on HIF-1α.^105^ Recently, FGF23 is also discovered to promote the osteogenic differentiation of BMSCs through endocrine mode.^106^ Overall, FGF signaling pathways are essential for coordinating osteogenesis and angiogenesis, with distinct FGFs exhibiting specific roles in these processes.

##### PDGF/platelet-derived growth factor receptor β (PDGFRβ) pathway

PDGFs consist of several subunits (A-D) that form homodimers or heterodimers, such as PDGF-AA, PDGF-BB, PDGF-DD, and PDGF-AB, through disulfide bonds. PDGFs bind to two receptors, PDGFRα and PDGFRβ. Among these, PDGF-BB and PDGF-DD exhibit high affinity for PDGFRβ, a type III receptor tyrosine kinase expressed on ECs, osteogenic lineage cells, and osteoclast precursors. PDGF binding to PDGFRβ occurs in a paracrine manner and activates downstream signaling pathways, influencing vascular invasion and bone formation. For instance, the PDGF-BB/PDGFRβ signaling pathway activates downstream Akt and extracellular regulated protein kinases (ERK1/2), promoting the proliferation and osteogenic differentiation of BMSCs.^107^ This pathway also regulates angiogenesis, which further enhances bone formation.^108^ Interestingly, the effects of PDGF-BB/PDGFRβ signaling on osteogenesis appear to be time-dependent. Wang et al. found that inhibition of PDGFRβ during the late stage of osteogenic induction enhanced the osteogenic differentiation of BMSCs, with both ERK1/2 and Smads involved in this process.^109^ The early stage of osteogenesis is primarily associated with cell proliferation and angiogenesis, while the late stage focuses on osteoblast differentiation and mineralization. Consequently, the PDGF-BB/PDGFRβ pathway regulates osteogenesis-angiogenesis coupling in a time-dependent manner.

In recent years, the discovery of type H and type E vessels has revealed their significant roles in osteogenesis-angiogenesis coupling.^110,111^ Type H vessels, in particular, are closely linked to bone homeostasis and osteogenesis. ECs of type H vessels promote osteogenesis and play critical roles in maintaining bone health.^111–115^ The PDGF/PDGFRβ pathway has been found to participate in this process. Osteoclast precursors can induce the formation of type H vessels by secreting PDGF-BB in a paracrine manner.^116^ Additionally, various bone-related factors can inhibit the differentiation of osteoclast precursors into OCs, thereby increasing PDGF-BB levels, which in turn upregulate type H vessel formation.^117,118^ Since type H vessels are strongly associated with osteogenesis, they provide a critical link between osteogenesis and angiogenesis, serving as an essential medium for intercellular communication among osteoclast precursors, type H vessel ECs, and other osteogenic cells. Moreover, ECs of type E vessels can transform into those of type H or type L vessels.^111^ The discovery of these vascular subtypes offers new insights into intercellular communication in osteogenesis-angiogenesis coupling, emphasizing the complexity and versatility of this process.

##### Other paracrine cytokines and pathways

In addition to the aforementioned paracrine signaling pathways, several other signaling molecules and related pathways also involved in osteogenesis-angiogenesis coupling through paracrine mechanisms.

IGF signaling pathway comprises IGF ligands (IGF-1 and IGF-2), IGF receptors (receptor of IGF-1 and IGF2, and insulin receptor), and IGF binding proteins. These components regulate cell growth, differentiation, and metabolism, and play a significant role in osteogenesis-angiogenesis coupling, particularly IGF-1.^119^ IGF-1 binds to its corresponding receptor, activating it through self-phosphorylation of its tyrosine kinase domain, which triggers downstream pathways such as MAPK and PI3K/Akt. Loss of IGF1 receptor results in impaired endochondral bone formation in OBs.^120^

The Hedgehog signaling pathway is a highly conserved intracellular signaling cascade involved in embryonic development, self-renewal, angiogenesis, and other functions. This pathway includes sonic hedgehog (SHH), Indian hedgehog, desert hedgehog, transmembrane receptors (patched and smoothened), and intranuclear factors like Glioma-associated oncogene homolog proteins, Fused, and suppressor of Fused. In classical SHH signaling, SHH binds to the patched receptor on recipient cells, releasing smoothened inhibition. This dissociation causes the suppressor fused to release Glioma-associated oncogene homolog proteins, which promotes the expression of target genes and induces intracellular effects.^121^ The Hedgehog pathway supports osteogenesis-angiogenesis coupling by promoting OB osteogenesis and EC angiogenesis in vitro,^122^ and enhancing bone formation via angiogenesis regulation.^123^ In vivo, inhibition of Hedgehog signaling suppresses bone formation and vascularization in stress fracture healing models.^124^

The Hippo/Yes-associated protein (YAP), another highly conserved pathway, regulates both osteogenesis and angiogenesis. YAP promotes MSC osteogenic differentiation while suppressing adipogenesis.^125^ Additionally, Hippo/YAP signaling pathway activates Cdc42, enhancing tip cell migration and filopodia formation, thereby regulating new vessel sprouting.^126^ Recent studies suggest that alpha calcitonin gene-related peptide acts as an upstream molecule that modulates Hippo/YAP signaling, promoting osteogenesis and angiogenesis.^127^

Slit guidance ligand (SLIT) 3, a secreted ECM protein, plays a crucial role in osteogenesis-angiogenesis coupling by interacting with ROBO receptors. Highly expressed in OBs, SLIT3 directly induces the formation of type H vessels.^128^ Loss of SLIT3 results in reduced type H vessel formation, leading to low bone mass and impaired fracture healing in mice.^129^

**Fig. 2:**
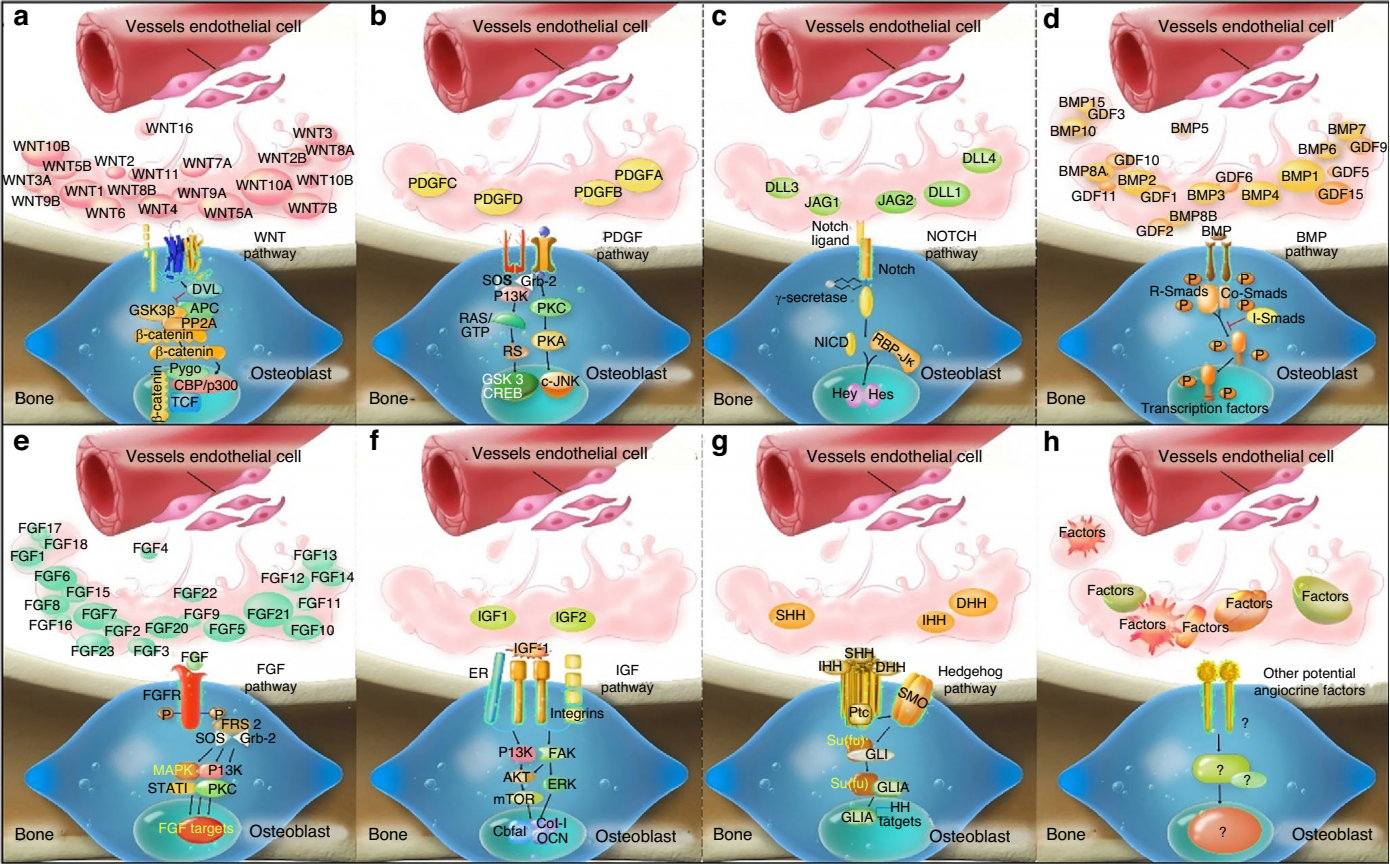
Key signal pathways involved in communication between bone and vascular cells. **a** The WNT signaling pathway and its ligands. **b** The PDGF signaling pathway. **c** The Notch signaling pathway. **d** The BMP Signaling pathway and its ligands. **e** The FGF signaling pathway. **f** The IGF signaling pathway. **g** The Hedgehog signaling pathway. **h** Other potential models of ECs factors regulating OBs. (Reproduced with permission^153^; Copyright 2020, Ivyspring International Publisher)

These pathways, along with other paracrine signaling molecules, are central to the cell communication involved in osteogenesis-angiogenesis coupling (Fig. 2). Further exploration of these pathways will deepen our understanding of cell communication and its applications in bone regeneration and vascularization.

#### Crucial juxtacrine signaling pathways

##### Notch pathway

Unlike most signaling pathways that operate via paracrine mechanisms, the Notch pathway relies on direct cell-cell contact. Both Notch receptors and their ligands, such as Delta-like ligand 4 (Dll4), are single-pass transmembrane proteins, enabling this unique mode of juxtacrine signaling.

The Notch pathway significantly impacts osteogenic cells, vascular cells, OCs, and the processes of osteogenesis and angiogenesis.^21^ Ramasamy et al.^115^ reported that Dll4-Notch signaling in bone ECs plays a unique role in promoting angiogenesis during osteogenesis, particularly through its involvement in type H vessels. This contrasts with the inhibitory effects of Dll4-Notch signaling on angiogenesis in other tissues. Specifically, Notch signaling in bone ECs upregulates VEGFR2, stimulating ECs proliferation and angiogenesis in postnatal long bones.

The Dll4-Notch pathway links vascular effects with direct influences on chondrocytes and OB lineage cells. On one hand, Notch signaling in ECs, activated through direct contact between adjacent ECs, triggers VEGF signaling, which guides the differentiation of tip and stalk ECs. These vascular responses subsequently influence osteogenesis. On the other hand, Notch signaling in ECs facilitates the secretion of osteogenic factors, such as Noggin (a BMP antagonist), into the bone microenvironment. These factors target osteoprogenitor cells (OPCs), OBs, and chondrocytes, thereby promoting bone formation through paracrine mechanisms.^130^ This links the vascular system with OPCs, OBs, and chondrocytes,^112^ demonstrating the significant role of NOTCH pathways in the osteogenesis and angiogenesis coupling through direct cell-cell contact communication mode.

##### Eph/Ephrin pathway

In addition to the Notch pathway, the Eph/Ephrin pathway is another key signaling mechanism facilitating direct cell-cell communication, distinguished by its bidirectional signaling capability. Both Eph receptors and their ligands, Ephrins, belong to the Eph protein family. Eph receptors are transmembrane proteins composed of an extracellular ligand-binding domain, a transmembrane domain, and an intracellular domain. Ephrins are divided into two subunits: EphrinA and EphrinB. EphrinA is anchored to the cell membrane via a glycosylphosphatidylinositol anchor, whereas EphrinB is a transmembrane protein with extracellular, transmembrane, and cytoplasmic domains.^131^ Previous studies have demonstrated the bidirectional signaling role of EphB4/EphrinB2 in mediating cell communication between OBs and osteoclast precursor cells.^132,133^ Beyond its role in bone cell communication, the Eph/Ephrin pathway also plays a significant role in angiogenesis. In vitro experiments have shown that the inhibition of the EphA2/EphrinA1 pathway suppresses the angiogenic ability of human umbilical vein endothelial cells (HUVECs), highlighting its importance in vascular development.^134^

## Cell communication of osteogenesis-angiogenesis coupling during normal bone development and maintenance

### Osteogenesis-angiogenesis coupling during normal bone development and maintenance

Bone development is a critical aspect of human growth, with osteogenesis-angiogenesis coupling playing a pivotal role in this process. Bone formation occurs through two primary mechanisms: intramembranous ossification and endochondral ossification. During these processes, OBs and chondrocytes secrete VEGFA and other signaling molecules to influence ECs, thereby regulating angiogenesis.^28,29,135^ The angiogenic activity of ECs is further linked to the establishment of primary and secondary ossification centers in the epiphysis.^136,137^

Bone development also involves the transformation of heterogeneous blood vessel subtypes. Type E (embryo-type) vessels are strongly associated with Osx^+^ OBs compared to type H vessels, and both are temporally and spatially confined during developing bone. Type E vessels dominate osteogenesis during embryonic and early postnatal periods of long bone development. However, genetic lineage tracing shows that type E vessels transition to type H vessels and subsequently to type L vessels during bone development.^111^ Type L vessels are essential for transporting hematopoietic cells, contributing to the formation of vascular niches required for myelopoiesis.^138–140^ These vascular transformations highlight the dynamic coupling of osteogenesis and angiogenesis, which plays a crucial role in the progression from bone growth to bone maintenance.

**Fig. 3:**
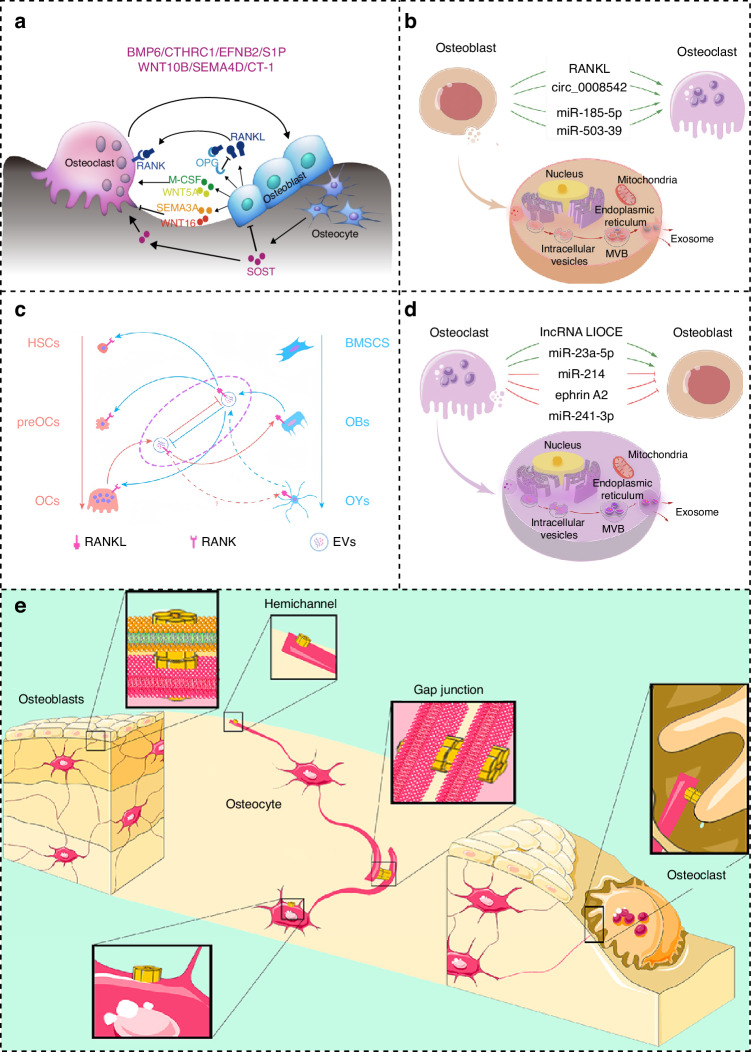
Communication between OBs and OCs. **a** Paracrine signaling molecules in OB-OC communication (Reproduced with permission^320^; Copyright 2018, Nature Portfolio). **b** Vesicular RANKL and miRNAs in OB-exos (Reproduced with permission^321^; Copyright 2022, Frontiers Media S.A.). **c** Vesicular RANK and RANK Reverse Pathway in OB-OC communication (Reproduced with permission^322^; Copyright 2019, BMC). **d** Membrane proteins and miRNAs in osteoclast-derived exosomes (OC-exos) (Reproduced with permission^321^; Copyright 2022, Frontiers Media S.A.). **e** Gap junctions between OBs, OCs, and Osteocytes (Reproduced with permission^323^; Copyright 2023, MDPI)

Bone maintenance (or homeostasis) is another essential process involving two key events: bone formation and bone resorption.^141^ The balance between these processes largely depends on the interactions between OBs and OCs, mediated by paracrine signaling molecules and exosomes (Fig. 3). Osteogenesis-angiogenesis coupling also contributes to this balance. For example, ECs and bone cells (such as OBs and hypertrophic chondrocytes) produce angiocrine and pro-angiogenic factors, including VEGF, to activate signaling pathways that regulate bone remodeling.^142^

The SLIT/ROBO pathway, a key regulator of type H vessel formation, not only promotes bone formation but also suppresses bone resorption, demonstrating its dual role in maintaining bone homeostasis.^143^ Type H vessels are particularly significant in this process, as their density is closely linked to Osx^+^ OPCs. This coupling is influenced by occlusal force through the mechanosensory PIEZO1/Ca^2+^/HIF-1α/SLIT3 axis, which highlights the mechanical regulation of bone homeostasis.^144,145^

Overall, the osteogenesis-angiogenesis coupling underpins the intricate coordination required for both bone development and long-term bone maintenance, emphasizing its critical role in skeletal health.

### Cell communication during normal bone development and maintenance

#### OB-EC communication

##### Signaling molecules in paracrine mode

**Fig. 4:**
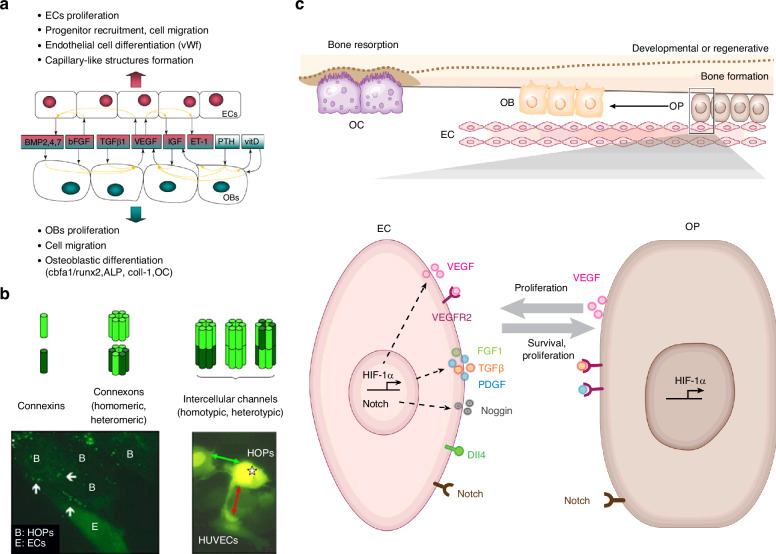
OB-EC communication. **a** Paracrine communication and soluble factors between OBs and ECs. **b** GJCs between human osteoprogenitor cells (HOPs) and HUVECs (Fig. 4**a** and **b** are reproduced with permission^2^; Copyright 2009, Cell Press). **c** Paracrine soluble factors and the Notch signaling pathways in direct-contact mode of the cell communication between OPs (i.e., OPCs) and ECs, regulating the differentiation of OPs to OBs (Reproduced with permission^130^; Copyright 2016, Annual Reviews Inc.)

Paracrine signaling is the predominant communication mode between OBs and ECs in osteogenesis-angiogenesis coupling due to the close proximity of these cells and the diversity of signaling molecules involved. Key pathways include the HIF-1α/VEGF axis, PDGF/PDGFRβ, and mechanistic target of rapamycin complex (mTORC) 1/CXCL9, along with other signaling molecules such as BMP2, IGF-1, and basic FGF (Fig. 4a).

***HIF-1α/VEGF axis***

HIF-1α/VEGF axis plays a pivotal role in regulating OB-EC communication during osteogenesis and angiogenesis coupling.^146,147^ VEGF, secreted by OBs and other bone-related cells, is regulated by HIF-1α expression, which increases under hypoxic conditions or external stimuli such as salidroside, ultrasound, and shock waves.^67,68^ Hypoxia-induced HIF-1α enhances VEGFA secretion in OBs, promoting angiogenesis in ECs via paracrine signaling. In osteoporotic conditions, reduced HIF-1α, VEGFA, BMP2, and Osx expression leads to impaired type H vessel formation and diminished bone regeneration. Interestingly, HIF-1α/VEGF exhibits age-dependent effects: while it promotes VEGF expression and angiogenesis in young BMSCs, it inhibits VEGF expression in aged BMSCs.^148–151^ These findings suggest that the HIF-1α/VEGF axis has a complex, age-dependent role in OB-EC crosstalk, warranting further research.

***PDGF/PDGFRβ pathway***

The PDGF/PDGFRβ is another important paracrine pathway influencing both vascularization^107^ and bone formation,^152^ bridging OB-EC communication. EC-derived PDGF-BB enhances the osteogenic effects of VEGF,^153^ upregulates VEGF expression via cAMP and protein kinase C pathways,^112,154^ and directly facilitates OB formation by interacting with perivascular cells that differentiate into osteogenic progenitors such as OPCs and OBs.^152,155^ Furthermore, PDGF-BB secretion by OBs and OCs promotes angiogenesis, highlighting its dual role in osteogenesis and angiogenesis coupling.^11,116^

***mTORC1/CXCL9 pathway***

OBs can also secrete inhibitory signals such as CXCL9, which negatively regulates EC angiogenesis by binding to VEGFA and preventing its interaction with VEGFR on ECs.^156^ CXCL9 secretion is regulated by mTORC1, a serine/threonine kinase complex that modulates osteoblast precursor differentiation via pathways like signal transducer and activator of transcription 3/p63/Jagged/Notch.^157,158^ While mTORC1 activity in OB precursors inhibits angiogenesis, mTORC1 in ECs influences angiogenesis through elongation, budding, and integration processes, often interacting with the HIF-1α/VEGF axis.^159,160^ Additionally, mTORC2, distinct from mTORC1, promotes angiogenesis through CXCL12/C-X-C chemokine receptor 4 signaling in ECs.^161,162^

***BMP2 and other signals***

BMP2 is crucial for bone fracture repair, functioning throughout the whole process of bone healing. Hypoxia and VEGF can upregulate BMP2 expression in ECs, which in turn stimulates OB formation at fracture sites.^64,82,83^ BMP2 synergizes with VEGF and TGF-β to promote osteogenic differentiation through the classical bone morphogenetic protein receptor-Smad signaling pathway.^84^

EC-derived endothelin-1 (ET-1) and IGF-1 also regulate OB activity. ET-1, commonly associated with angiogenesis, influences OBs by modulating Toll-like receptor signaling and cytokine expression such as interlcukin (IL)-18.^163,164^ IGF-1, a component of bone matrix IGF-1, secreted by various bone-related cells, promotes MSC migration and OB differentiation,^165,166^ highlighting its pro-osteogenic role.

***Other angiocrine factors***

Additional EC-derived signals, such as those from FGF and Hedgehog pathways, also contribute to OB-EC communication. For example, EC-derived FGF2 and FGF11 promote osteogenesis and angiogenesis, while Hedgehog signaling regulates bone formation and vascularization.^43,127,128^ However, the underlying mechanisms of these pathways require further exploration.

In summary, paracrine signaling between OBs and ECs involves diverse pathways and molecules that play complementary roles in osteogenesis and angiogenesis coupling, with key factors like HIF-1α/VEGF, PDGF-BB, mTORC1/CXCL9, BMP2, ET-1, and IGF-1 leading the coordination. Further investigation is necessary to clarify the complex interactions of these pathways in bone regeneration and maintenance.

##### EVs-mediated indirect OB-EC communication

Exosome-based vesicle is also a vital indirect OB-EC communication mode and serve a bridge for material exchange between the two cells. Current researches mainly focus on the regulatory effects of exosomes-derived miRNAs on OBs and ECs. A previous study showed that miRNA let-7f-5p from mineralizing OB-derived exosomes promotes angiogenesis of ECs through the dual specificity phosphatase 1/ERK1/2 signaling pathway.^167^ Aging OBs also express certain specific miRNAs that regulate the activities of ECs, among which miR-214-3p targets the gene L1CAM to promote aging and apoptosis of ECs, and inhibit their proliferation and migration.^168^ Conversely, exosomes originating from ECs can also act as messengers delivering miRNA to regulate OBs. It has been demonstrated that miR-92b-3p, carried by microvascular ECs-derived exosomes, inhibits MC3T3-E1 cells differentiation into mature OBs by targeting ETS-like transcription factor 4.^169^ Additionally, ECs-derived exosomes can reverse glucocorticoid-induced suppression of bone formation by inhibiting osteoblast ferroptosis.^170^

Besides miRNAs, exosomes also transport functional proteins to participate in OBs-ECs communication. Mature OBs-derived exosomes carry the matrix metallopeptidase 2 which acts on ECs after internalization and thus promotes angiogenesis through the VEGF/ERK1/2 signaling pathway.^171^ The exosomes, enhanced with VEGF through plasmid transfection (i.e., VEGF-EC-exos), transport VEGF to MSCs and promote their differentiation into OBs by activating the MAPK/ERK pathway.^172^ Due to the specific characteristic, exosomes can be used as the carriers of signaling molecules (functional proteins and miRNAs) and thus applied in bone tissue engineering. Mi et al. designed a multifunctional hydrogel for exosome/drug delivery by incorporating artificially produced EC-exos containing miR-26a-5p to regulate the differentiation of OBs/OCs, thereby promoting bone fracture repair.^173^

Furthermore, studies indicate that EVs can transmit Notch signaling to affect the process of angiogenesis and the morphology of blood vessels. Dll4 carried by exosomes can bind to Notch receptors on EVs to activate the Notch pathway, and other EVs can also transmit Notch receptors.^174^ Moreover, there is a report that exosome-mediated Notch ligands can induce the retraction of capillary sprouts.^175^ All these studies indicate that the Notch pathway can also function through EVs, affecting the process of angiogenesis and the morphology of blood vessels. It is hypothesized that in the coupling of osteogenesis and angiogenesis, some Notch ligands might activate the Notch pathway in this manner to transmit signals to more distant cells. However, no study has yet confirmed the definite influence of Notch pathway on OBs-ECs communication through EVs.

##### Potential role of gap junction in direct OB-EC communication

In addition to OBs-ECs communication mediated by direct contact between membrane-bound ligands and receptors, gap junction may be another potential direct communication mode between OBs and ECs. Currently researches mainly focus on GJCs between ECs and BMSCs, OPCs (See Fig. 4b in Ref. ^2^), rather than OBs. GJCs composed of Cx43 exist between human bone marrow stromal cells and HUVECs.^14^ Inhibiting the synthesis of Cx43 can reduce the influence of co-cultured HUVECs on the differentiation of human bone marrow stromal cells into OBs, and this inhibitory effect can be further disseminated among cells through signaling molecules permeating the GJCs.

Regarding the substances transmitted through GJCs, the signaling molecules affecting osteogenesis-angiogenesis coupling through other communication mode, may also be transmitted via gap junctions or adhesion junctions.^176^ Fan et al.^177^ found that miRNA-200b could be transferred through GJCs between BMSCs and HUVECs. TGF-β promotes the transfer of miR-200b from BMSCs to HUVECs, leading to decrease of miRNA-200b expression and increase of VEGFA expression in BMSCs. VEGFA can exert pro-osteogenic effects through autocrine mechanism, while miRNA-200b transmitted into HUVECs through gap junctions downregulates the expression of angiogenic factors, inhibiting HUVEC migration and the formation of vessel-like structures. This indicates that miRNAs can act as “messengers” delivered through GJCs to participate in intercellular communication. With the aid of laser confocal microscopy, Grellier et al.^176^ found that mitochondria can be transferred from BMSCs to rat lung microvascular ECs through Cx43-composed GJCs. Based on above researches, it can be speculated that signaling substances, such as miRNAs and mitochondria, might also be transferred through gap junctions between OBs and ECs, generating a more complex communication mode. However, there is still lack of related researches currently.

##### Ligand-receptor mediated direct-contact communication

Interaction between membrane-bound ligands and receptors is the main direct contact mode for cell communication and is crucial for cell proliferation, growth, and metabolism. Among the signaling pathways involved in this mode, the Notch signaling pathway plays a significant role in OBs-ECs communication. In 2014, Ramasamy et al.^115^ discovered that the Dll4-Notch signaling pathway promotes angiogenesis in the skeletal vascular system, a finding that is contrary to the inhibitory effects observed in other areas of angiogenesis research. Additionally, they found that endothelial cell-specific activation of the Notch pathway in bone can facilitate the proliferation of H-type ECs and the secretion of pro-osteogenic factors like Noggin, which acts on OBs to enhance osteogenic activity.^112,130^ The Notch signaling pathway is pivotal in regulating the biological activities of OBs. OBs can express various Notch ligands and receptors, thereby activating the Notch pathway through direct binding of Notch ligands and receptors on the cell membranes. This direct cell-cell communication regulates osteogenesis-angiogenesis coupling (See Fig. 4c in ref. ^130^). This mode has been demonstrated in co-culture models of osteogenic lineage cells and ECs.^178^ Various factors, such as G-protein-coupled receptor kinase 2-interacting protein 1^179^ and miRNAs,^180^ can regulate intercellular communication between osteogenic lineage cells and ECs by influencing the Notch pathway, thus affecting physiological processes like angiogenesis, osteogenesis, and bone healing.

##### ECM-derived factors in OB-EC communication

Besides above soluble and membrane-binding signaling molecules, ECM-derived molecules such as TGF-β1 also play a role in OBs-ECs communication. TGF-β1 is abundantly embedded in bone matrix and is released during bone resorption and remodeling. TGF-β1 can regulate VEGF secretion in OBs through various signaling pathways, including p38/MAPK, stress-activated protein kinase/c-Jun N-terminal kinase, p44/p42 MAPK, and Smad2/3, thereby affecting endothelial cell angiogenesis. Other signals, such as wnt3a, may also affect this process and promote TGF-β1-induced VEGFA secretion in OBs via the canonical Wnt signaling pathway.^41,181^ Additionally, TGF-β1 acts on ECs and decreases expression of the membrane-bound Notch signaling pathway receptor Notch1 and VEGFR2 involved in angiogenesis.^42^ The Notch pathway plays a crucial role in distributing characteristics of tip cells and stalk cells in ECs during angiogenesis.^182,183^ The exogenous TGF-β1-induced reduction in Notch1 expression disrupts the allocation towards tip cell characteristics on the matrix gel, while the shedding of VEGFR2 affects the reception of VEGF signals by ECs, ultimately inhibiting angiogenesis. Hence, factors in the bone matrix, represented by TGF-β1, can influence osteogenesis-angiogenesis coupling through regulating of OBs-ECs communication (Fig. 4c).

#### OC-EC communication

**Fig. 5:**
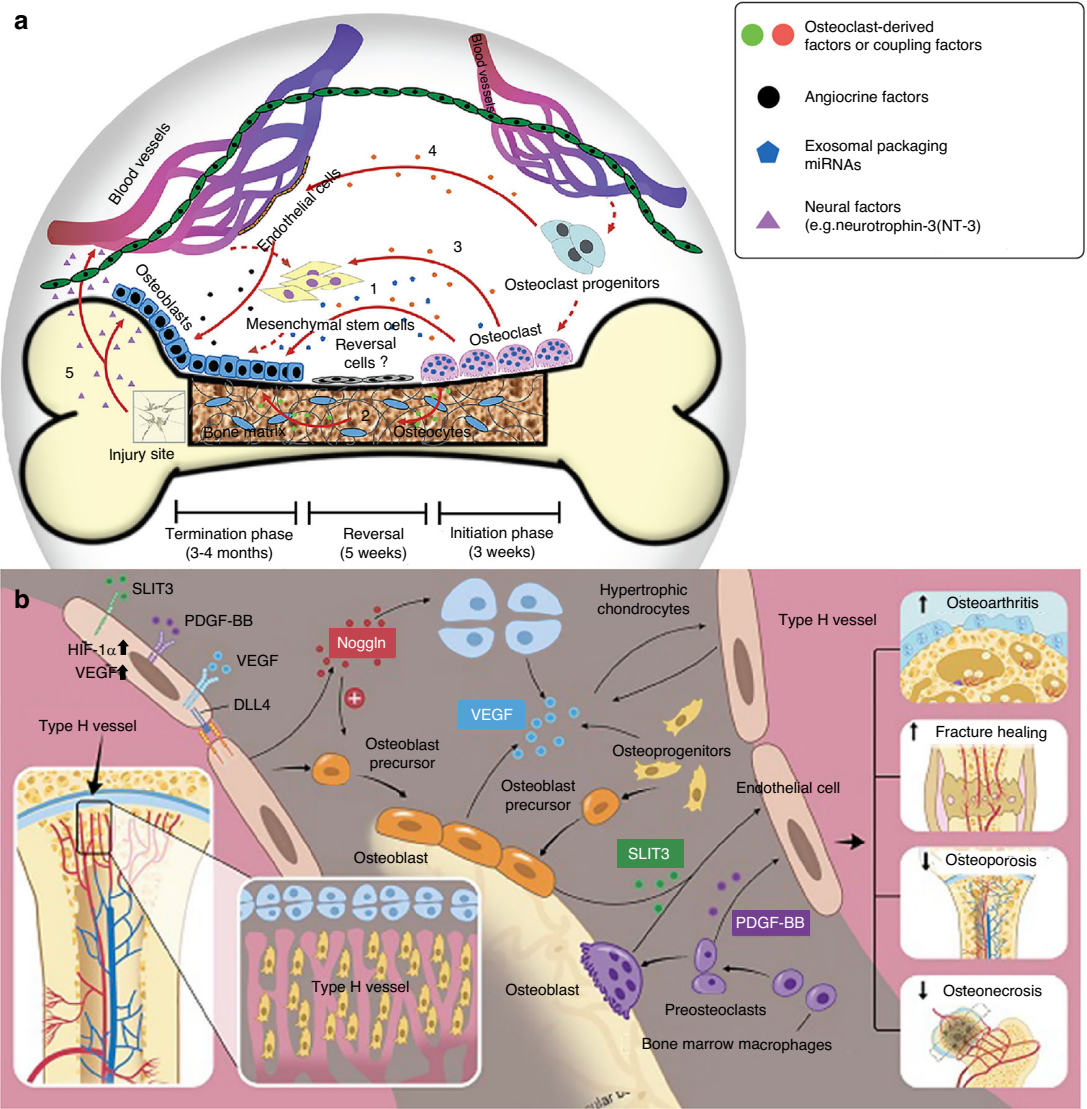
OC-EC communication. **a** Different modes of cell communication in the conversation of OCs, OBs, and ECs (Reproduced with permission^324^; Copyright 2018, John Wiley and Sons). **b** Cell communication of type H ECs with osteoclast and its precursor (Reproduced with permission^112^; Copyright 2020, Ivyspring International Publisher)

In addition to OBs, OCs also play a crucial role in bone biology and actively interact with ECs to regulate osteogenesis-angiogenesis coupling. Various modes of signaling transmission, including paracrine signaling and EVs, facilitate OC-EC communication, activating key pathways that influence both osteogenesis and angiogenesis (Fig. 5a).

##### Paracrine signals in OC-EC communication

Paracrine signals between OCs and ECs involves key molecules such as HIFs, VEGF, and PDGF-BB. Hypoxia triggers the differentiation of human peripheral blood mononuclear cells into OCs, a process closely linked to HIF signaling in osteoclast precursors. Early studies suggested that this differentiation under hypoxia is driven by HIF-1α-mediated signaling. However, recent research distinguishes the roles of HIF-1α and HIF-2α: HIF-1α is primarily associated with osteoclast bone resorption, whereas HIF-2α predominantly promotes the differentiation of human CD14⁺ monocytes into OCs.^184–186^

VEGF also affects OCs, as VEGFC and VEGFD can bind to receptors on OCs membranes, enhancing RANKL-induced monocytes-to-OCs differentiation.^65^ VEGFA, the most potent angiogenic member of the VEGF family, also regulates the OCs formation.^187^ It has been shown that hypoxia and RANKL upregulate HIF-1α in mature OCs via the NF-κB pathway, which in turn enhances VEGFA secretion.^188^ This VEGFA secretion promotes angiogenesis in ECs and stimulates them to secrete osteogenic factors such as BMP-2. Additionally, VEGFA induces ECs to express semaphorin 3A (Sema-3A) in a dose-dependent manner.^189^ Sema-3A inhibits osteoclast formation, promotes osteoblast differentiation, and can induce apoptosis in osteoclast precursors such as RAW264.7.^190,191^ Through these actions, the HIF-1α/VEGF axis emerges as a central pathway linking OC-EC communication.

Type H vessels, reported by Kusumbe and Ramasamy^110^ in 2014, are a specialized vascular subtype found at the end of the growth plate and in subperiosteal regions. These vessels communicate with osteogenic cells to form an osteo-angiogenic coupling vital for bone formation and remodeling.^112^ Osteoclast precursors secrete PDGF-BB, which promotes the formation of Type H vessels and aggregation of EPCs.^116^ Studies have shown that inhibiting PDGF-BB (e.g., using Zoledronate) reduces EPC angiogenic function and osteoblast differentiation.^192^ Furthermore, proteins such as G protein-coupled receptor kinase 2 interacting protein-1 regulate PDGF-BB secretion from osteoclast precursors, thereby controlling Type H vessel formation.^193^ Thus, PDGF-BB is indispensable for OC-EC communication and the osteogenesis-angiogenesis coupling (Fig. 5b).

##### Exosomes-derived miRNA in OC-EC communication

The role of EVs-mediated signal transmission between OCs and ECs is increasingly recognized, with miRNAs as key cargo molecules. Recently, Wang et al.^194^ found that the expression of miR-146a in exosomes from mechanically compressed OCs is downregulated. Adiponectin is a target of miR-146a, and the downregulation of miR-146a in OC-exos leads to an increase in adiponectin expression in HUVECs, thereby promoting angiogenesis. This suggests that modulating the cargo delivery of OC-exos through specific mechanical stimuli or osteo-angiogenic signals can influence angiogenesis and bone remodeling.

Exosomes from OCs not only affect ECs but also regulate OCs and their precursors. Song et al.^36^ found that EC-exos are more effective on bone targeting and osteoclast function inhibition compared to exosomes from OCs or BMSCs. EC-exos carrying miR-155 can be internalized by bone marrow macrophages (BMMs), delivering miR-155 to alter BMM morphology and inhibit their differentiation into OCs. This miR-155-mediated communication between ECs and BMMs inhibits osteoclast formation, as confirmed by exosomal miRNA sequencing. Blocking miR-155 expression in BMMs reverses the inhibitory effect of EC-exos on osteoclastogenesis. Furthermore, exosomes from other angiogenesis-related cells also regulate OCs. Exosomes derived from EPCs promote osteoclast formation through lncRNA-MALAT1, while pericyte-derived exosomes inhibit monocyte-to-osteoclast differentiation by suppressing the tumor necrosis factor associated factor 3-mediated NF-κB signaling pathway.^195^ Currently, EC-exos are being explored as vehicles for delivering signaling molecules or drugs, such as miRNAs, to regulate osteoclast and osteoblast activity in bone fracture repair.^173^

##### Direct contact: the potential role in OC-EC conversation

The Eph/Ephrin pathway is crucial for angiogenesis and serves as a bidirectional signaling pathway that mediates cell communication through direct contact. Its effects on ECs depend on the specific subunits involved—EphrinA or EphrinB. EphrinA1/EphA2 regulates EC migration, influencing both speed and direction. Overexpression of EphrinA1 reduces EC proliferation, whereas gene silencing enhances proliferative activity.^196^ EphrinB2 influences endothelial cell budding and migration by interacting with VEGFR3 and its downstream signaling pathways, affecting vasculature formation.^197^ Additionally, recent research indicates that EphrinB2/EphA4 activates ECs, increasing monocyte adhesion.^198^ Given that monocytes can differentiate into OCs and serve as osteoclast precursors, this suggests that the Eph/Ephrin pathway may facilitate direct OC-EC communication, contributing to the coupling of osteogenesis and angiogenesis. Although most research on Eph/Ephrin regulation of angiogenesis has focused on tumor biology, its role in osteogenesis-angiogenesis coupling remains to be fully elucidated.

#### An integrated signaling cascade in OB-OC-EC cross-communication

**Fig. 6:**
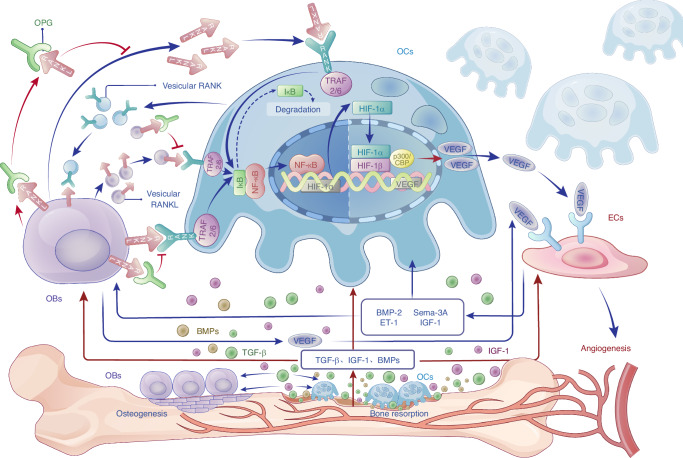
An integrated signaling cascade in OB-OC-EC cross-communication and osteogenesis-angiogenesis coupling

Osteogenesis and its coupling with angiogenesis are complex processes necessitating involvement of various bone-related cells in bone microenvironment. The signaling exchange among these cells usually presents a multiple-directional crosstalk mode rather than a dual-way interaction as mentioned above, thus forming a sophisticated network cross-communication. To illustrate the crosstalks among OBs, OCs, and ECs, an integrated and multiple-directional signaling cascade is proposed here (Fig. 6). In this cascade, RANKL from OBs is delivered to OCs either in a paracrine manner, through exosomes, or via direct cell-cell contact. RANKL then binds with membrane receptor RANK on the surface of OCs and their precursors and activates downstream signal cascades through NF-κB, which eventually induce an increase of HIF-1α expression.^188^ Cytoplasm HIF-1α enters the nucleus and binds with HIF-1β. The formed heterodimer acts on target gene-VEGF and upregulates its expression. VEGF from OCs is released into bone microenvironment and acts on ECs to induce angiogenesis. In addition, OBs can secrete VEGF directly to regulate angiogenesis of ECs.^67,68^ In response to VEGF stimulation, ECs also secrete pro-osteogenic factors such as BMP-2,^64^ ET-1, IGF-1,^165^ and Sema-3A,^189^ which can reversely act on OBs and OCs and regulate their differentiation and function.^84,166,190,199,200^

As the competitor of RANKL, soluble OPG from OBs can bind with membrane-bound RANKL, soluble RANKL and vesicular RANKL to prevent the activation of corresponding signaling pathway and thus inhibit differentiation and function of OCs.^201,202^ Additionally, OCs can engage with OBs through vesicular RANK, which interacts with membrane-bound RANKL on the OBs surface and regulates osteoblast differentiation via the RANK Reverse pathway.^203,204^ The activity of mature OCs and resorption of bone tissue result in the release of TGF-β1,^75^ BMPs^72,73^ and IGF-1^205,206^ from bone matrix, all of which participate in regulating the activities of these three types of cells.

This multiple-direction signaling cascade only partially elucidates the cross-communication among OBs, OCs and ECs, as numerous signal pathways and cell communication modes are involved in this process, rather than the cascade proposed above only. In a recent study, a tri-culture model was established to evaluate the dynamic interplay among OBs, OCs, and ECs.^207^ This model induced different intracellular effects compared to bi- and mono-cell cultures, which may provide a more effective way to simulate osteogenesis-angiogenesis coupling in vivo. Therefore, it is essential to integrate different signal pathways and cell communication modes as a whole to construct an integrated cross-communication network among OBs, OCs, and ECs to highlight the mechanism of osteogenesis and its coupling with angiogenesis.

#### Immune- and other bone-related cells in cell communication of osteogenesis-angiogenesis coupling

##### Macrophages

Macrophages represent a crucial element of the immune system, displaying remarkable adaptability in their response to diverse microenvironments and stimuli. Macrophages facilitate bone formation by influencing both OBs and OCs, supporting OC function and promoting bone anabolism.^208–210^ M0 macrophages differentiate into two main types: the M1 and M2, each with distinct functional attributes. M1 macrophages are distinguished by the secretion of pro-inflammatory cytokines, including tumor necrosis factor-α (TNF-α) and interleukin (IL)-1, which facilitate the activation of the RANKL/RANK signaling pathway, thereby promoting OC-mediated bone resorption. Furthermore, M1 macrophages express inducible nitric oxide synthase, leading to ROS production,^208^ which further amplifies inflammatory response. In contrast, M2 macrophages secrete anti-inflammatory factors, such as IL-10 and IL-4, thus promoting osteogenesis and inhibiting osteoclastogenesis. They also produce TGF-β, which plays a pivotal role in osteogenesis of MSCs.^208^

Macrophages, particularly the M2 subtype, are integral to bone angiogenesis. They secrete large amounts of PDGF-BB, a critical mediator that orchestrates effective angiogenesis by recruiting pericytes and MSCs. These recruited cells initiate vascular sprouting and help stabilize new blood vessels. Moreover, activation of the HIF-1α signaling pathway can induce a TNF-α–positive phenotype in macrophages, potentially enhancing the angiogenic functions of cell lines such as RAW264.7.^208^ During angiogenesis, macrophages form bridges between ECs and promote new vessel branch formation. They secrete VEGF and other cytokines that stimulate endothelial regeneration and contribute to side branch growth, especially under hypoxic conditions. Additionally, perivascular macrophages in small vessels help regulate osmotic responses.^211^

##### Neutrophils

Neutrophils, another crucial component of the immune system, also participate in the communication processes that couple osteogenesis and angiogenesis. In the early stages of rheumatoid arthritis, neutrophils migrate into the joint cavity, instigating an inflammatory response. They exhibit an increased capacity to form neutrophil extracellular traps, which are implicated in activating synovial fibroblasts and modulating immune responses.^212^ Neutrophils communicate with target cells by transferring cytosolic lipids and proteins via microvesicles. This intercellular communication activates genes that regulate chondrocyte anabolism, promoting ECM accumulation and enhancing cartilage protection.^212^ In acute inflammation triggered by bacterial lipopolysaccharide, neutrophils enhance their glycolytic activity, leading to increased production and release of lactate. Lactate mobilizes inflammatory bone marrow neutrophils by acting on the GPR81 receptor expressed by ECs, which locally increases vascular permeability. Furthermore, it promotes rapid neutrophil mobilization from the bone marrow by elevating levels of neutrophil-attracting chemokines (CXCL1 and CXCL2) and increasing the release of granulocyte colony-stimulating factor.^213^ Single-cell RNA sequencing of periodontal lesions in mice has revealed that neutrophils interact with OBs during periodontitis-induced bone loss. Periodontal lesions exhibit significant neutrophil infiltration, and these neutrophils communicate with OBs through cytokine secretion. Notably, neutrophil-derived oncostatin M effectively induces RANKL expression in primary OBs. Conversely, deletion of the oncostatin M receptor in OBs markedly ameliorates periodontitis-induced bone loss.^214^ These findings underscore that neutrophils regulate osteogenesis by signaling with OBs, chondrocytes, and ECs. This crosstalk reveals a novel mechanism underlying the coupling of osteogenesis and angiogenesis, highlighting the multifaceted roles of immune cells in bone biology.

##### BMSCs

BMSCs constitute a heterogeneous population comprising distinct subgroups with unique molecular and functional properties. Notably, BMSCs derived from the metaphysis (mpMSCs) and the diaphysis (dpMSCs) exhibit intrinsic differences. The proliferation, colony-forming capacity, and multipotency of BMSCs both in vivo and in vitro are predominantly associated with PDGFRα⁺β⁺ Hey1⁺ mpMSCs rather than dpMSCs or reticular cells. The fate of BMSCs is regulated by PDGFRβ signaling and the transcription factor Jun-B. Overexpression of PDGF-BB increases the number of Osx⁺ OPCs, inhibits the formation of perilipin⁺ adipocytes, and promotes the development of type H vessels and arterioles in the bone vasculature.^215^ During human MSC osteogenesis, the secretion of DJ-1 (i.e., parkinsonism-associated deglycase 7) is significantly enhanced. Released DJ-1 can stimulate vascularization in the endothelium via activation of FGFR1 signaling.^216^ Direct application of DJ-1 to the culture medium is sufficient to stimulate human MSC differentiation into OBs, mediated by FGFR1 activation and VEGF secretion.^216^ Additionally, BMSC-derived exosomes accelerate the proliferation and migration of ECs and OBs, promote angiogenesis and osteogenesis, and thus facilitate fracture healing. As intercellular communicators, these exosomes activate the HIF-1α/VEGF and BMP-2/Smad1/Runx2 signaling pathways, enhancing osteogenesis and fracture repair.^217^ EPCs have been demonstrated to enhance the osteogenic differentiation of BMSCs and facilitate ectopic bone formation. The combined use of EPC-BMSC in irradiated bone defects provides sources of the MSC-osteoblast lineage and the vascular lineage, thereby enhancing bone healing in the tibia of irradiated rats.^218^

Recent research indicates that EC glycolysis regulates bone vascularization and mitigates osteoporosis through H3K18la lactylation in BMSCs. Lactate secreted by ECs mediates paracrine crosstalk between ECs and BMSCs. Clinical data suggest that elevated lactate levels correlate with increased bone mineral density, and that raising blood lactate levels through exercise may help prevent osteoporosis.^219^

##### Perivascular cells/pericytes

Skeletal capillaries can be classified into two distinct types based on their morphology, molecular markers, and functions: type H and type L. ECs of type H vessels support local vascular growth and provide crucial microenvironmental signals to perivascular osteogenic progenitor cells. The proliferation of type H ECs is positively regulated by endothelial Notch and HIF-1α signaling pathways. The abundance of CD31^hi^/Emcn^hi^ ECs serves as an important indicator of skeletal vascular growth and osteogenic capacity. A reduction in type H vasculature, along with a decrease in osteogenic progenitor cells, may explain bone loss associated with aging and suggests potential therapeutic strategies to improve osteogenic capacity in older adults.^110^

Notch activity in ECs promotes Noggin expression in osteogenic progenitor cells, facilitating osteoblast differentiation. Noggin also aids in chondrocyte maturation and hypertrophy. VEGF secreted by osteogenic progenitors and chondrocytes promotes skeletal angiogenesis, while SLIT3 produced by mature OBs and PDGF-BB from pre-OBs enhance the expression of type H ECs.^144^ Under normal conditions, PDGFRβ⁺ perivascular cells are found surrounding type H blood vessels and actively participate in osteogenesis. In pathological states, a reduction in HIF-1α/PDGF-BB/PDGFRβ signaling between ECs and perivascular cells leads to the detachment of PDGFRβ⁺ cells from type H vessels. This separation disrupts endothelial–perivascular cell coupling, contributing to vascular injury, bone loss, and fat accumulation as these cells transition to a lipogenic phenotype.^220^

Pericytes exhibit MSC-like properties, including pluripotency, self-renewal, immunomodulation, and a multifaceted role in tissue repair. Characterized by the expression of specific surface markers such as PDGFR and CD146 (and being negative for CD34 and CD45), pericytes secrete high levels of various growth and differentiation factors through paracrine effects, thus accelerating tissue repair both in vitro and post-transplantation.^221^ Pericytes play key roles in neointimal formation and sprouting angiogenesis via signaling molecules such as PDGFR-β, TGF-β, VEGF and angiopoietin-1. They are critical for the formation and sprouting of new blood vessels. TGF-β1 is essential for maintaining endothelial cell–pericyte interactions, as it is closely associated with the contractility that fixes pericytes to the surface of ECs. In the absence of TGF-β1, pericyte loss can lead to uncontrolled endothelial proliferation and excessive angiogenesis at injury sites.^222,223^

Ang1 stimulates ECs to release factors that recruit pericytes, including TGF-β1 and PDGF-B. Endothelial cell-derived PDGF-B promotes the migration and proliferation of pericytes expressing PDGFR-β. Moreover, the differentiation of mesenchymal cells into pericytes when co-cultured with ECs is associated with increased VEGF production by mesenchymal cells, potentially mediated by TGF-β. This VEGF expression by differentiated pericytes supports endothelial cell survival and microvessel stability.^223^

## Cell communication of osteogenesis-angiogenesis coupling in fracture healing and regeneration

### Key phases of fracture healing and osteogenesis–angiogenesis coupling

Bone regeneration consists of a well-orchestrated series of biological events, including the intercellular cell communication of various cells and intracellular signal transduction of plentiful signaling pathways, while the fracture healing is the most canonical form of bone regeneration in the clinical setting.^224^ Osteogenesis of fracture healing is realized through either intramembranous bone formation or endochondral bone formation. In intramembranous bone formation, MSCs directly differentiate into OBs, which then mineralize the ECM to the eventual osteogenesis. It mainly occurs in the development of flat bones such as maxilla and mandible, and in rigidly fixed fractures with minimal fracture gaps and in fractures within the bone metaphysis. While during the endochondral bone formation, MSCs priorly differentiate into chondrocytes, and the chondrocytes build a novel cartilage matrix layer and latterly converted into woven bone undergoing further ossification. Endochondral bone formation mainly occurs in the development of long bones, and in fractures located in the diaphysis with less mechanical stability and large fracture gaps achieve bone repair through this way.^225–228^

Regardless of the type of bone formation, fracturing healing is a complex process involving early inflammatory response, angiogenesis, osteogenic differentiation and ossification, and the osteogenesis-angiogenesis coupling plays an indispensable role in this process.^229^ During the early inflammatory response period, hematoma formation at the fracture site creates a relatively hypoxic environment that upgrades the expression of HIF-1α, which in turn influences its downstream signaling molecules such as VEGF to promote subsequent angiogenesis and osteogenesis in the next healing period.^230^ BMPs-secreting MSCs is recruited to the early hematoma, preparing for subsequent differentiation into chondrogenic or osteogenic linages.^231^ Immune system is also actively involved in this period and macrophages polarized to M1 phenotype to clean up debris in the fracture site. In the next angiogenic period, angiogenesis is the key to cartilage or soft callus formation. ECs invade into fracture sites, constructing vascular network through angiogenesis to achieve the vascularization of injury sites, and this is accompanied by the invasion and recruitment of OPCs.^110^ OPCs differentiate into chondrocytes for endochondral bone formation to form soft callus or directly differentiate into OBs to perform intramembranous bone formation, constructing hard callus. In this period, macrophages switch from M1 to M2 phenotype with anti-inflammatory characterization, promoting tissue healing and reducing inflammatory bone resorption.^232^ Adequate vascularization ensures enough supplement of oxygen and nutrients for the subsequent osteogenic differentiation and ossification, and ECs also secret signaling factors during the fracture healing process to interact with MSCs, OBs, and macrophages. Therefore, the osteogenesis-angiogenesis coupling is a significant feature in fracture healing, and it is characterized by complex cell-cell communication among bone-related cells, ECs, and immune cells, especially macrophages.

### Crosstalk between macrophages, ECs, and bone-forming cells during fracture repair

The intercellular communication among OBs, OCs, ECs, macrophages and other related cells in osteogenesis-angiogenesis coupling, are well introduced in sections “OB-EC communication”, “OC-EC communication”, “An integrated signaling cascade in OB-OC-EC cross-communication” and “Immune- and other bone-related cells in cell communication of osteogenesis angiogenesis coupling”. Most of these events are applicable in fracture healing and therefore they will not be discussed more. The following will focus on communication of macrophages with ECs and MSCs, which shows specificity and is critical for osteo-angiogenesis coupling in fracture healing.

#### Cell communication of macrophages with ECs

Cell communication does exist between the macrophages and ECs. A recent study has shown that macrophages are crucial for maintaining the sinusoidal and arterial-like populations of primary mouse bone marrow ECs in vitro, thereby protecting the native transcriptomic profiles and functional heterogeneity of the ECs.^233^ Co-culture of macrophages with human outgrowth ECs and human primary OBs produces more vascular tubes compared with OBs-ECs bi-culture system. And both the concentration of VEGF in supernatants and secretion of inflammatory factors such as IL-6, IL-8, TNF-α from the macrophages are increased in this tri-culture system.^234^ This indicates that macrophages communicate actively with ECs through paracrine or other modes to regulate vessel formation and thus influence the osteogenesis-angiogenesis coupling. In addition, fracture healing is a dynamic process from inflammation to tissue regeneration, during this process macrophages also experience the phenotype transition from pro-inflammatory M1 to anti-inflammatory M2. Therefore, the influence of macrophages and their communication with ECs are closely associated with their phenotype which may be determined by fracture healing process.

The paracrine pro-angiogenic factors actively participate in macrophages-ECs communication. Upregulation of activating transcription factor 4 expression in alveolar bone-derived macrophages leads to increasement of VEGFA, and knockout of this transcription factor induces the decrease of M1 markers and VEGFA expression in these macrophages, and eliminates the gap of angiogenic capability of these macrophages with those from long bone.^235^ On one hand, macrophages could regulate angiogenesis through pro-angiogenic factors such as VEGFA, and macrophage polarization may affect this effect. On the other hand, the ECs-derived pro-angiogenic factors also play a role in regulating macrophages and their polarization. Implantation of VEGF-containing vascular grafts can promote the switching of infiltrating monocytes into M2 macrophages, while in the heparin-containing grafts monocytes are inclined to convert into M1.^236^

The angiogenesis-associated signaling pathways also involves in macrophages-ECs communication. M1 macrophages could promote ECs migration through secreting molecules such as VEGF and TNF-α.^237^ While M2 macrophages promote angiogenesis directly.^238^ Moreover, it is discovered that M2 macrophages promote the angiogenesis, vascular permeability of ECs through the HIF-1α/VEGFA signaling pathway.^239^ A recent study has shown that M2 macrophages promote ECs angiogenesis in retinal neovascularization through CXCL12/VEGF signaling axis.^240^ Other molecules or signaling pathways may also regulate macrophages polarization and their communication with ECs in bone fracture healing, but the underling mechanisms need to be further elucidated.

Exosomes also mediate macrophages-ECs communication and regulate bone regeneration. The EC-exos released under the stimulation of inflammatory cytokines can activate macrophages through the MAPK/NF-κB signaling pathway.^241^ They also regulate macrophages polarization through the containing miRNAs and lncRNAs to regulate bone regeneration.^242,243^ In contrast, macrophages-derived exosomes could also regulate ECs. Exosomes secreted by M2 macrophages promote angiogenesis both in vivo and in vitro, and the pro-angiogenic effect on HUVECs is associated with miRNA-5106.^244^

Additionally, the classification of M1 and M2 macrophages is primarily based on their roles in regulating inflammation rather than their capacity to promote tissue regeneration. While M2 macrophages are generally regarded as more conductive to tissue regeneration, this effect is largely attributed to their anti-inflammatory properties rather than a direct regnerative function. Recently, a new subpopulation of activated macrophages is identified as CD301b macrophages, although there is some overlap with M2 macrophages that highly express CD206. The CD301b population is more specific to the regenerative microenvironment and can induce bone formation.^245^ It also actively participates in osteogenesis-angiogenesis coupling and VEGF is a key factor to regulate CD301b macrophages-ECs communication.^246,247^ Although the underling mechanisms remains to be further explored, it is certain that CD301b macrophage population plays an important role in fracture healing due to its pro-regenerative characterization.

#### Macrophage-MSC communication

In the process of fracture healing, macrophages complete the transformation from pro-inflammatory M1 to anti-inflammatory M2. M1 macrophages are dominant in the early inflammatory healing stage, while M2 macrophages secret growth factors to support osteogenic differentiation of MSCs thereafter, thus promoting the bone formation. Meanwhile, studies demonstrate that M1 macrophages and the inactivated M0 macrophages also communicate with MSCs.^232^

Champagne et al. have proved that the inactivated J774a.1 murine macrophage cell line can increase the alkaline phosphatase activity in human MSCs, and this effect is mediated by the BMP2, indicating that the interaction of M0 macrophages with MSCs may participate in the fracture healing.^248^

The role of M1 macrophages on the regulation of MSCs and fracture healing is controversial. Study has shown that the M0, M1 and M2 macrophages all enhance ECM mineralization when co-cultured with mouse MSCs, and the M1 macrophages manifest the most significant effect. This process is highly associated with cyclooxygenase-2/prostaglandin E2 signaling pathway.^249^ The M1 macrophages may secrete signaling factors that recruit MSCs into the bone fracture sites, and MSCs undergo osteogenic differentiation and eventual ossification with the influence of M2 macrophages. Cell communication between M1 macropahges and MSCs is also supported by a study in which the BMSC-derived exosomes inhibit polarization and secretion of pro-inflammatory factors of M1 cells, thus promoting rotator cuff tendon-bone healing.^250^ But there are also studies indicating that M1 macrophages inhibit bone formation in fracture healing through cell-cell communication with MSCs. For instance, M1-derived exosomes transfer miR-222 to induce the apoptosis of MSCs.^251^ The M1 macrophages-triggered inflammatory response is the premise of the normal healing of bone. However, prolonged inflammation is detrimental to the fracture healing due to excessive bone resorption.^228,252,253^ The controversial effects of M1 macrophages on MSCs reflect their dual action on fracture healing which is determined mainly by different healing stages of bone fracture.

There is indeed communication between M2 macrophages and MSCs. In a three-dimensional co-culture model of macrophages and human MSCs, the MSCs inhibit M1 macrophage and promote M2 polarization, and the MSCs co-cultured with both macrophages exhibit stronger osteogenic differentiation ability compared to cultured alone.^254^ In the tibial mono-cortical bone defect, the macrophage scavenger receptor 1 activates the PI3K/Akt signaling pathway to promote osteogenic differentiation of BMSC and the M2 polarization.^149^

M2 macrophages and MSCs can communicate with each other through paracrine mode. Murine bone marrow polarized M2 macrophages could promote osteogenesis of murine bone marrow MSCs in an indirect transwell co-culture system, and the expression of TGF-β, VEGF, and IGF-1 are all significantly increased in M2 macrophages. Consequently, it is reasonable to speculate that the growth factors upregulated in M2 macrophages play a role in the bone formation. However, the molecules participating in cell communication between M2 macrophages and the MSCs in a paracrine mode remains unclear.^255^ Recently, a novel study reveals that the IL-10 may be a candidate of mediator in the paracrine communication between M2 macrophages and MSCs, in which the nanohydroxyapatite particle-treated M2 macrophages promote the osteogenesis of MSCs in an IL-10 dose-dependent manner.^256^ Moreover, the exosomes may also participate in communication between M2 macrophages and MSCs. Mechanical force-induced macrophage exosomes could promote BMSCs osteogenesis through the ubiquitin carboxyl-terminal hydrolase isozyme L3, which targets the Smad1.^257^ While the miRNA of MSCs-derived exosomes could switch the macrophage polarization from M1 to M2 in the spinal cord injury.^258^ The specific mechanism of exosome-mediated cell communication between M2 macrophages and MSCs during fracture healing remains to be further illustrated.

### Cell communication between bone and vessels in calvarial fracture

Unlike long bones, recent studies suggest that osteogenesis and angiogenesis in calvarial fracture healing may not follow the tightly coupled dynamics observed in other skeletal regions. Using advanced longitudinal intravital multiphoton microscopy, Bixel et al. revealed that the collective invasion of OPCs in calvarial fractures is not simultaneously accompanied by early angiogenic sprouting. In their findings, endothelial Notch and VEGF signaling only influenced vascular regeneration without significantly affecting ossification by OBs, arguing for a potential decoupling of osteogenesis and angiogenesis in calvarial fracture healing.^259^

In long bones, such as femurs, the invasion of OPCs typically follows closely behind the sprouting blood vessels into the avascular callus, with OPCs and blood vessels comigrating and forming collagen bundles that intersect with vascular protrusions in a finger-like arrangement. However, in calvarial fractures, OPCs and OBs initially form a multicellular sheet on the bone surface and begin their collective invasion only after sufficient vascularization of the lesion area is achieved. Although the timing of OPC and vascular invasion differs between these bone types, the osteogenesis-angiogenesis coupling is not solely defined by simultaneous events.^260,261^

Despite the apparent temporal separation, cell communication between bone and vascular cells persists during calvarial fracture healing. Molecules secreted by ECs and OBs influence each other when OBs arrive at the vascular network. Similar to long bone fractures, Osx^+^ OBs and newly formed bone matrix remain proximal to microvessels, enabling paracrine interactions between OBs and ECs.^262^ In addition, various signaling pathways mediate the regulation of fracture healing through osteogenesis-angiogenesis coupling. For instance, YAP, a key component of the Hippo signaling pathway, promotes osteogenic commitment of MSCs while also regulating type H vessel formation. However, YAP suppresses HIF-1α expression in bone ECs, potentially impairing fracture healing.^263^ Although endothelial Notch and VEGF signaling appear to have limited impact on ossification in calvarial fractures, other pathways may mediate OB-EC communication and influence the process indirectly.

## Cell communication of osteogenesis-angiogenesis coupling in other bone-related diseases

### Osteoporosis

Osteoporosis results from an imbalance between bone resorption and bone formation, leading to reduced bone mass, compromised bone quality, and impaired vascular formation. Studies have shown that the reactive oxygen species-mediated HIF-1α/p53 axis plays a bidirectional regulatory role in the osteogenesis-angiogenesis crosstalk in long bones.^264^ Guan et al. demonstrated that targeting the activation of the Ca^2+^ channel protein Piezo1 in a mouse osteoporotic bone defect model enhanced the nuclear accumulation of YAP and β-catenin, facilitating osteogenesis-angiogenesis coupling during the early stages of bone healing and accelerating bone reconstruction.^265^

BMSCs are pivotal in osteoporosis management, as they enhance calcium deposition, collagen synthesis, and angiogenesis by secreting various cytokines and growth factors. CD31^hi^ Emcn^hi^ type H-positive vessels, crucial for oxygen delivery and vascularization, stabilize OPCs and support BMSC-mediated bone regeneration in osteoporosis.^208^ Activation of the PI3K/Akt signaling pathway, which regulates β-catenin, promotes osteogenesis-angiogenesis coupling in BMSCs.^266^ Similarly, activation of the Wnt/β-catenin pathway—key for development and homeostasis—stimulates bone formation and the generation of CD31^hi^ Emcn^hi^ type H-positive capillaries in fracture sites.^267,268^ These findings suggest that targeted activation of the Wnt/β-catenin pathway may serve as a promising therapeutic strategy for combating osteoporosis.

Beyond bone cells such as BMSCs, the osteoporosis microenvironment features significant immune dysregulation, particularly an imbalance between T regulatory cells and T helper cells (Th). Th2 cells exhibit protective effects against osteoporosis progression by producing cytokines such as IL-4, IL-5 and IL-13, which inhibit osteoclastogenesis and promote parathyroid hormone production, thereby reducing the RANKL/OPG ratio and reversing bone loss. Conversely, Th17 cells exacerbate osteoclastogenesis by secreting pro-inflammatory cytokines, including IL-1, IL-6, IL-17, RANKL and TNF. An imbalance in the T regulatory/Th cell ratio is a key factor in osteoporosis pathogenesis, as T regulatory cells may lose regulatory capacity and transition into Th17 cells in osteoporotic conditions.^208^ CD4^+^ T helper cells also play a critical role in angiogenesis. Th2 cells secrete growth factors that enhance angiogenesis, a mechanism leveraged in bone regeneration scaffolds to attract Th2 cells and promote vascularization. Furthermore, CD4^+^ T cells recruit macrophages to enhance angiogenesis.^208^

### Osteoarthritis

The development of osteoarthritis is primarily driven by the immune system’s response to an inflammatory environment. IL-3, secreted by T lymphocytes, has been shown to increase the expression of chondrocyte-specific genes such as sex-determining region of Y chromosome-box transcription factor (Sox) 9 and type IIa collagen. In contrast, IL-1β downregulates Sox9 and type IIa collagen expression. Notably, IL-3 inhibits the expression of matrix metalloproteinases induced by IL-1β and TNF-α, thereby mitigating cartilage degradation. IL-6, secreted by activated T lymphocytes, exhibits dual effects on chondrocytes: it protects cartilage by inhibiting metalloproteinase production, yet it suppresses proteoglycan synthesis, thereby reducing type IIa collagen production and exacerbating IL-1β-mediated cartilage degradation. Additionally, activated T cells produce TGF-β, which plays a key role in cartilage remodeling by enhancing Sox9 expression, facilitating chondrocyte differentiation, and reducing IL-6 receptor expression in chondrocytes.^212^

In osteoarthritis pathogenesis, type H-positive vessels and MSCs form a positive feedback loop, driving abnormal subchondral bone formation. Type H-positive vessels supply MSCs, which contribute to aberrant bone formation, while MSCs enhance angiogenesis through the MAPK pathway. Inhibiting this interaction can alleviate osteoarthritis.^269^ Single-cell RNA sequencing has identified various cell types involved in temporomandibular joint osteoarthritis. For instance, M1 macrophages promote inflammation, angiogenesis, and innervation, while CD31^+^ ECs facilitate bone mineralization. Chondrocytes express genes related to inflammation, angiogenesis, and ossification, such as genes of epidermal growth factor receptor and integrin alpha-5.^270^ Additionally, increased VEGF expression is associated with the catabolic activity of chondrocytes and synovial cells, correlating with disease severity.^271^ Co-culture of HUVECs, dermal fibroblasts, and BMSCs has been shown to facilitate osteogenic and angiogenic niches, promoting osteogenesis and angiogenesis.^272^ Moreover, osteoclastogenesis can be inhibited by modulating NF-κB and MAPK signaling pathways. Downregulation of PDGF-BB further alleviates subchondral bone resorption and angiogenesis in mice, rescuing cartilage degeneration.^273^

### Osteonecrosis

Osteonecrosis is driven by increased osteoclast activity and impaired angiogenesis, disrupting interactions between immune and bone cells. Drug-related osteonecrosis of the jaw, often associated with anti-resorptive drugs like bisphosphonates, is a rare but severe condition. Zoledronic acid has been shown to enhance osteoclastogenesis by inducing IL-6-mediated upregulation of RANKL through the janus tyrosine kinase 2/signal transducer and activator of transcription 3 pathway.^274^ Additionally, bisphosphonates promote the release of pro-inflammatory cytokines such as IL-6, TNF-α and IL-1β, while suppressing IL-10 secretion, creating a pro-inflammatory microenvironment. This environment not only impairs NK cell-mediated cytotoxicity but also reduces the regenerative capacity of MSCs, further inhibiting wound healing and contributing to drug-related osteonecrosis of the jaw.^275,276^ MSC-derived EVs show potential in mitigating bisphosphonate-induced damage by reducing senescence in OBs and fibroblasts, promoting angiogenesis and bone regeneration.^277^

Radiotherapy for tumors can lead to radiation-induced osteonecrosis, characterized by necrosis of osteocytes, endothelial damage, and the creation of a hypoxic, low-cellular environment. Radiation promotes osteoclast differentiation by increasing RANKL expression and disrupting immune-skeletal interactions through persistent lymphopenia and altered immune cell phenotypes.^278^ MSCs can restore angiogenesis and promote tissue repair by stimulating endothelial progenitor cell differentiation, enhancing tube formation, and activating angiogenic growth factors, offering a promising therapeutic strategy for radiation-induced damage.^279^

Hormone-induced osteonecrosis, often caused by prolonged glucocorticoid use, results in metabolic abnormalities, microvascular disruption, and trabecular bone necrosis. Glucocorticoids impair angiogenesis and osteogenesis by reducing the expression of key factors like HIF-1α, PDGF-BB and VEGF, and by increasing Notch-1 expression, which inhibits osteogenic differentiation and disrupts osteogenesis-angiogenesis coupling.^280^ Reduced p-Akt expression is another hallmark of glucocorticoid-induced osteonecrosis, with therapeutic upregulation of p-Akt enhancing osteoblast proliferation, angiogenesis, and reducing apoptosis through pathways such as Akt/Runx2.^281,282^

MSC therapies have shown promise in addressing osteonecrosis by promoting angiogenesis and bone repair. MSCs can secrete factors like COL6A2 to activate the PI3K/Akt signaling pathway, enhancing endothelial migration and angiogenesis. Additionally, the use of composite scaffolds with MSCs and EPCs further supports osteogenesis, angiogenesis, and reduces adipogenesis, facilitating osteonecrosis repair.^283,284^

## Clinical therapeutic approaches based on cell communication in osteogenesis–angiogenesis coupling

The intricate interplay between osteogenesis and angiogenesis via cellular communication opens promising avenues for innovative clinical therapies targeting bone-related diseases and enhancing bone tissue engineering. By harnessing specific signaling pathways that mediate interactions among OBs, ECs, MSCs, immune cells, and pericytes, researchers can design targeted interventions to promote coordinated bone and blood vessel regeneration. Below, we outline key clinical approaches based on cell communication in osteogenesis-angiogenesis coupling.

### Therapies based on intercellular signal transmissions

Cell communication mechanisms facilitate signal transmission between donor and recipient cells. These processes can be leveraged to deliver signaling molecules that regulate osteogenesis-angiogenesis coupling, offering therapeutic benefits for bone-related diseases.

#### Paracrine growth factor-based therapies

Growth factors play a critical role in mediating communication between bone and vascular cells, facilitating both osteogenic and angiogenic processes. Therapeutic strategies often focus on the delivery of these key paracrine growth factors to stimulate OB differentiation, bone formation, and EC function. For example, BMPs, particularly BMP-2 and BMP-7, are frequently utilized due to their strong osteoinductive properties.^64^

Growth factors can be embedded in biomaterials such as hydrogels and implanted into bone defects. Once implanted, these factors release signals to nearby bone cells and ECs in a paracrine manner, promoting angiogenesis and osteogenesis, and ultimately facilitating the repair of bone defects.^285^ External stimuli—such as ultrasound, temperature changes, or light—can be applied to control the timing and order of growth factor delivery. This approach helps prevent rapid degradation of the factors, ensuring sustained release for optimal therapeutic effect. For instance, VEGF is essential for promoting angiogenesis by inducing EC proliferation and migration. It also indirectly supports osteogenesis by enhancing the vascular supply to bone-forming regions.^67,68^ Ramesh et al. designed a triple growth factor delivery system by inserting VEGF, PDGF, and low-dose BMP-2 into hydrogel.^286^ The simultaneous release and continuous delivery of VEGF and PDGF in this system promotes angiogenesis, allowing new bones to regenerate up to 60% of the intact bone at low doses of BMP-2. Lack of clear does-dependent modes of BMP-2 to bone regeneration has incurred the application of high-dose BMP-2 in complex bone fractures such as bone-muscle combined injuries clinically to acquire adequate bone regeneration,^287^ but high-dose BMP-2 also induces serious adverse effects such as heterotopic bone formation.^288^ The co-delivery of growth factors elevates the therapeutic effects of low-dose BMP-2 to substitute the therapy of high-dose BMP-2. This approach is particularly valuable for serious bone fractures combined with muscle injuries, such as segmental bone defects with volumetric muscle loss, where effective bone regeneration is crucial.

##### Exosome-based therapies

Beyond traditional paracrine signaling, exosomes serve as crucial mediators of cell communication in osteogenesis–angiogenesis coupling. Secreted by various cell types, exosomes carry bioactive molecules—such as miRNAs, proteins, and lipids—that regulate both osteogenic and angiogenic processes. This capacity makes exosomes promising novel carriers for therapeutic substances aimed at enhancing bone regeneration. Exosomes can be engineered to carry specific miRNAs or signaling molecules, enhancing their therapeutic potential and offering a personalized approach to bone repair. As naturally occurring vesicles, exosome-based therapies are minimally invasive and highly biocompatible, reducing the risk of immune rejection or adverse reactions. MiR-26a-5p is known to promote the osteogenic differentiation of MC3T3-E1 cells.^289^ Mi et al. engineered mouse endothelial cell exosomes (from Bend.3 cells) by incorporating a CD9-HuR fusion protein to efficiently load and transfer miRNA-26a-5p.^173^ This strategy protected the miRNA during transit and ensured its effective delivery to OBs, thereby increasing the expression of osteogenic-related genes. By leveraging engineered exosomes as carriers for specific therapeutic molecules, exosome-based therapies offer a novel, efficient, and biocompatible approach to treating bone-related diseases, ultimately enhancing bone repair and regeneration through precise regulation of osteogenesis and angiogenesis.

### Therapies targeting intracellular signal transduction

#### Targeting signaling pathways for therapies

Intracellular signal transduction is a pivotal aspect of cell communication in osteogenesis–angiogenesis coupling. Successful signal transduction within a recipient cell relies on intact signaling pathways. Hence, pharmacologically targeting components of these specific pathways offers a strategic means to promote tissue regeneration in bone-related diseases.

As essential activators of signaling pathways, ligands and receptors represent critical therapeutic targets. Modulating their expression or activity can stimulate key pathways involved in osteogenesis and angiogenesis. For instance, manipulating the Notch and Wnt/β-catenin pathways has shown promise in treating bone diseases.^290^ Notch signaling plays a complex role by regulating both bone formation and blood vessel growth. Pharmacological modulation of this pathway can significantly impact cell communication and tissue repair. Activation of Notch1 signaling in OPCs has been reported to enhance fracture healing.^291^ Conversely, targeted deletion of the Dll4 ligand in ECs can impair early fracture healing.

The pro-osteogenic effects of Notch signaling may vary with age. For example, loss of Nicastrin— a component necessary for Notch receptor activation—in aging skeletal stem cells has been found to increase bone mass, contrary to expectations. This highlights the importance of considering age-related changes when designing therapies targeting Notch pathways and suggests potential strategies to mitigate bone loss in elderly patients.^292^

Activation of the Wnt/β-catenin pathway supports both osteoblast differentiation and endothelial cell function, making it a valuable therapeutic target.^293^ Biallelic nonsense mutations in Wnt ligand genes can cause osteoporosis due to diminished Wnt/β-catenin signaling. Thus, regulating Wnt ligands to enhance this pathway is crucial for osteoporosis treatment. Various methods aim to stimulate the Wnt/β-catenin pathway. For instance, electroacupuncture has been shown to increase the expression of Wnt3α and β-catenin, positively influencing osteogenesis in postmenopausal osteoporosis models.^294^

#### MiRNA and gene therapies

Non-coding RNAs, particularly miRNAs, are key regulators of signaling pathway molecules involved in osteogenesis–angiogenesis coupling. By directly targeting intracellular components of these pathways, miRNAs modulate downstream signal transduction to influence both bone formation and vascular development. For instance, miR-150-5p promotes the formation of type H vessels targeting the Pi3k/Akt signaling pathway by regulating the downstream Sox2.^295^ Yang et al. also discovered that the depletion of the miRNA-497-195 cluster in ECs of mice decreases the angiogenesis of type H vessels and bone mass. The study further demonstrated that this miRNA cluster maintains stability of HIF-1α and supports Notch signaling activity by targeting genes such as P4HTM (Prolyl 4-hydroxylase, transmembrane) and Fbxw7 (F-box and WD-40 domain protein).^180^ Overexpression of the miRNA-497-195 cluster can mitigate age-related declines in type H vessels and bone mass, highlighting its potential as a therapeutic target for age-related osteoporosis. Additionally, various other miRNAs have been identified that could serve as therapeutic targets for bone-related diseases due to their regulatory impact on critical signaling pathways.^296,297^

Non-coding RNA regulatory effects on signaling pathways can be influenced by N6-methyladenosine methylation. Changes in N6-methyladenosine methylation status can alter bone-related activities such as bone resorption, thus indirectly affecting osteogenesis–angiogenesis coupling.^30^ Histone modifications represent another layer of epigenetic regulation that can influence osteogenic differentiation and signaling pathways. While recent studies have shown that histone modifications of osteogenesis-related genes significantly promote the differentiation of human MSCs, the precise links between these epigenetic changes, pro-osteogenic signaling pathways, and their mechanisms remain to be fully elucidated.^298,299^

These insights into miRNA functions, N6-methyladenosine methylation, and histone modifications open up promising avenues for genetic and epigenetic therapies. By targeting crucial signaling molecules or their regulators within pro-osteogenic or pro-angiogenic pathways, novel treatments or medicines can be developed to effectively regulate osteogenesis–angiogenesis coupling. This could lead to improved strategies for treating osteoporosis, enhancing fracture healing, and managing other bone-related disorders.

### Clinical applications of cell communication in osteogenesis–angiogenesis coupling

#### Biomimetic scaffolds and tissue engineering

Adequate vascularization is crucial for effective osteogenesis in bone defect sites, making cell communication that regulates angiogenesis vital in bone tissue engineering.^2^ Researchers leverage this intercellular dialogue to enhance osteogenesis–angiogenesis coupling in bone grafts and scaffolds, thereby improving graft survival and bone defect reconstruction.

Signaling molecules that mediate osteogenesis–angiogenesis coupling can be incorporated into biomaterials like hydrogels. These hydrophilic polymeric networks can be loaded with bone cells or signaling factors to promote vascularization and bone tissue formation at defect sites, and enhance recovery from excessive bone resorption in conditions such as periodontitis.^300–303^

Some bioactive element ions influence cell activities. Strontium enhances osteogenesis of OBs and angiogenesis of ECs.^304^ Zinc imparts antibacterial properties to implants.^305^ These ions can be integrated into bioactive ceramics, glasses, or scaffolds. Ion-doped bioceramics and bioactive glass scaffolds (e.g., cobalt-doped) have been used clinically to promote both bone formation and angiogenesis, facilitating wound healing and bone regeneration.^306,307^ Hybrid scaffolds combining hydrogels and bioactive materials enable programmed delivery of signaling factors for controlled osteogenesis–angiogenesis coupling.^302^

##### Cell-based therapies

Transplantation of stem or progenitor cells is a well-established method to stimulate bone and vascular regeneration, leveraging their inherent ability to differentiate and communicate within the osteogenesis–angiogenesis network. As multifunctional cells, MSCs can differentiate into OBs and perivascular cells, directly contributing to bone formation. They also secrete paracrine factors and exosomes that regulate osteogenic and angiogenic processes.^308^ The mechanisms of MSC therapy mainly depend on the osteogenic differentiation ability of MSCs. Studies have shown that the transplanted allogenic mouse MSCs eventually differentiate into OBs to directly regulate osteogenesis and promote fracture healing.^309^ Moreover, MSCs are a part of cell communication network in osteogenesis-angiogenesis coupling, consequently MSCs secrete paracrine molecules and exosomes to regulate osteogenesis of bone cells or angiogenesis of ECs.^310,311^ MSCs interact with the immune system; osteo-immunomodulatory materials can modulate inflammatory responses (e.g., via IL-17/ferroptosis pathways), enhancing MSC therapy effectiveness, particularly in cranial or mandibular defects.^312^

The methods of stem cell therapy include injection and transplantation of exogenous stem cells, and the grafted MSCs differentiate into OBs to promote bone repair. It is reported that 7 days after fracture may be the optimal time to inject BMSCs to promote fracture healing.^313^ Recently, the co-coculture strategy in which MSCs co-cultured with other progenitor cells has become a hot topic in cell-based therapies for bone regeneration.^314,315^ As the progenitor cells with the ability to differentiate into ECs, EPCs contribute directly to neovascularization and indirectly support osteogenesis by improving vascularization at the injury site. The synergistic interaction between EPCs and MSCs, mediated by juxtacrine and paracrine signaling, enhances bone regeneration. This approach has been applied to repair large segmental femoral defects using engineered scaffolds.^316,317^ Multicellular hybrid stem cell therapies combining various progenitor cell types hold promise for treating complex bone fractures and defects, leveraging enhanced intercellular communication to drive coordinated osteogenesis and angiogenesis.

By integrating biomimetic scaffolds, bioactive materials, and advanced cell-based therapies, clinical applications targeting cell communication in osteogenesis–angiogenesis coupling are paving the way for improved strategies in bone regeneration and repair.

## Conclusion and perspective

Osteogenesis is a crucial process for bone remodeling, fracture healing and bone defect repair, intricately coupled with new blood vessel formation both temporally and spatially. The interaction between osteogenesis and angiogenesis is mediated by complex cell communication networks involving various bone-related cells, signaling molecules, and pathways. In this article, we thoroughly review advances in cell communication within osteogenesis-angiogenesis coupling over the past decade, focusing primarily on the bidirectional and multidirectional interactions between OBs and ECs through different communication modes and associated signal pathways. Additionally, we discuss the cell communication in bone-related contexts such as the normal bone fracture healing and other pathological bone microenvironment.

Numerous signal transmission modes, including gap junction, tight junction, adhesion junction, paracrine, synaptic/neurotransmitter transmission, facilitate cell-cell communication among bone cells and ECs, serving as bridges for osteogenesis-angiogenesis coupling. A comprehensive understanding of these intercellular signal transmission modes and signal pathways, as discussed in this review, can broaden our knowledge on bone biology and inspire new strategies for treating bone-related diseases such as the drug delivery mediated by EVs and hydrogels embedded with growth factors in bone tissue engineering.

Despite significant progress in understanding cell communication among bone-related cells in osteogenesis–angiogenesis coupling, many aspects remain poorly understood. For instance, the direct contact communication between OC lineage cells and ECs, as well as the specific molecules within EVs that regulate osteogenesis and its coupling with angiogenesis, are not fully elucidated. The molecular mechanisms governing bone and vascular cell communication in calvarial fracture healing remain unclear, necessitating future studies to explore whether angiogenesis and osteogenesis interact through signaling pathways beyond the NOTCH and HIF-1α signaling. Further research is essential to elucidate these processes and identify potential therapeutic targets for the prevention and treatment of bone-related diseases.
